# Identification and Management of a Novel Brown Spot Disease in Plums (*Prunus salicina* Lindl.)

**DOI:** 10.3390/plants15030369

**Published:** 2026-01-24

**Authors:** Yaqi Luo, Yanhui Yang, Liguo Huang, Changyun Liu, Xinrui Du, Lulu Guo, Haoyue Ma, Meimei Long, Shanshan Li, Shanzhi Wang, Xianchao Sun, Guanhua Ma

**Affiliations:** Chongqing Key Laboratory of Plant Disease Biology, College of Plant Protection, Southwest University, Chongqing 400715, Chinasunxianchao@163.com (X.S.)

**Keywords:** *Colletotrichum nymphaeae*, identification, management, first report

## Abstract

Plum (*Prunus salicina* Lindl.), belonging to the genus *Prunus* in the *Rosaceae* family, is one of the most widely cultivated deciduous fruit trees globally. Plums are renowned for their round, sweet fruits, which are rich in a variety of bioactive compounds and are deeply loved by consumers. However, in 2021, alarming reddish-brown–dark brown sunken lesions appeared on the fruits of Qingcui plums in Wanzhou, Chongqing, China. The pathogens were identified as *Colletotrichum nymphaeae*, *Fusarium sulawesiense*, and *Fusarium pernambucanum*. The present study further describes the growth patterns and pathogenic differences of these strains in different environments, elucidating their infection mechanisms and pathogenic characteristics; these findings provide a theoretical basis for the efficient management of plum brown spot disease. Additionally, we determined that fluazinam is the most effective control agent against the plum brown rot caused by these pathogens. Notably, this study is the first to document plum brown spot disease induced by *C. nymphaeae* in China. These findings are intended to provide a vital theoretical framework for the scientific management and control of plum brown spot; furthermore, they underscore the necessity of proactive prevention strategies in agricultural settings.

## 1. Introduction

Plum (*Prunus salicina* Lindl.) holds a distinguished status among globally cultivated deciduous fruit trees, recognized for its nutritional richness encompassing vitamins, minerals, soluble sugars, organic acids [1], polyphenols, and their derivatives [2]. This esteemed fruit exhibits notable antioxidant properties and free radical scavenging capabilities [3,4], offering a plethora of health benefits including antioxidant and anti-inflammatory effects, potential in colon cancer and breast cancer prevention, weight management, and cognitive enhancement [5,6,7,8]. Despite the escalating cultivation and production of plums, the proliferation of various plum diseases poses substantial threats to both fruit quality and yield, thereby impeding the progress of the plum industry [9].

Among the predominant afflictions affecting plums, notable mentions include brown rot, bacterial canker, gummosis, plum brown spot, leaf blight, and red spot [9]. Particularly noteworthy is plum brown spot, a prevalent malady inflicting harm upon both fruits and foliage. Upon fruit infection, distinctive brown sunken lesions ensue, subsequently evolving into small black conidia discs, with the potential emergence of orange-red mucilaginous conidia under elevated humidity conditions [10,11]. The primary etiological agents responsible belong to the *Colletotrichum* genus, encompassing species such as *Colletotrichum gloeosporioides*, *C. nymphaeae*, *C. foriniae*, and *C. siamense*. *Colletotrichum*, a globally distributed plant pathogenic fungus, poses a significant threat to various plant species, both monocotyledonous and dicotyledonous, earning it the eighth position among the top ten plant pathogenic fungi globally in 2012 [12,13]. Within this genus, there are 14 compound species, including the *C. acutatum* species complex, *C. boninense* species complex, and *C. gloeosporioides* species complex [14], distinguished notably by the presence of brown conidia dispersed or aggregated on host surfaces or beneath the cortex [15]. In 2004, *Colletotrichum gloeosporioides* was isolated and identified from brown plum anthracnose in Changsha, Zhangjiajie, and other areas of Hunan Province [16]. In 2011, the causative agent of European plum fruit anthracnose in the production park of the Chinese Academy of Agricultural Sciences in Liaoning Province was identified as *C. acutatum* [17]. In 2021, Wang Qun [18] and others identified the pathogen of half-red plum anthracnose in Suijiang County as belonging to the *C. gloeosporioides* complex and *C. siamense* complex. Internationally, in 2019, South Korean researchers [19] isolated 24 strains of anthracnose fungi from diseased Japanese plum fruits and identified the pathogens as *C. gloeosporioides*, *C. nymphaeae*, *C. foriniae*, and *C. siamense* using morphological and phylogenetic analyses, with *C. nymphaeae* not previously reported in domestic plums.

Furthermore, plum leaf spot emerges as another prevalent ailment, characterized by gray-green lesions on leaves transitioning to gray-brown, and eventually to gray-white with slight rings. Fruit damage manifests as brown, slightly sunken spots. Recent reports from Meishan City, Sichuan Province, document plum leaf spot induced by *Fusarium* species, including *F. sulawesiense*, *F. pernambucanum*, and *F. citril*. *Fusarium*, a ubiquitous plant pathogenic fungus, poses a global menace, causing wilting, necrosis, root rot, stem rot, fruit rot, and even impacting animals and humans [20]. With over 500 reported species [21], Fusarium typically produces two types of spores, large conidia, and small conidia, with many species capable of chlamydospore production. However, *Fusarium* infection of plum fruits remains relatively underreported.

In 2021, a significant incidence of plum fruit brown rot was observed within plum plantations located in Wanzhou, Chongqing. This study aims to shed light on plum fruit brown rot, identifying its causative agents as *C. nymphaeae*, *F. sulawesiense*, and *F. pernambucanum*. Through a meticulous biological analysis, the infection characteristics of plum brown rot induced by these pathogens are delineated, alongside the determination of optimal preventive and control measures, thereby providing a theoretical framework for the scientific management of plum brown rot.

## 2. Results

### 2.1. Field Symptoms of Plum Brown Spot

**Figure 1:**
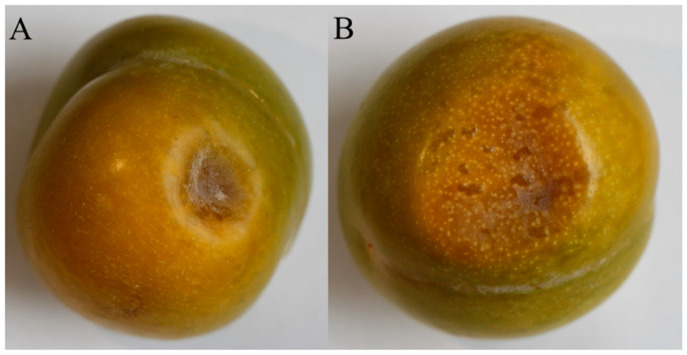
The symptom of plums. (**A**) Early symptoms of plum brown spot disease on plum fruits. (**B**) Late symptoms of plum brown spot disease on plum fruits.

In 2021, diseased plum fruits were collected in Wanzhou District, Chongqing, China, at an average altitude of 504 m. The fruits exhibited symptoms characterized by reddish-brown–dark-brown sunken spots (Figure 1), which indicated the presence of a slight mold layer and a soft texture.

### 2.2. Identification of the Isolated Strains

**Figure 2:**
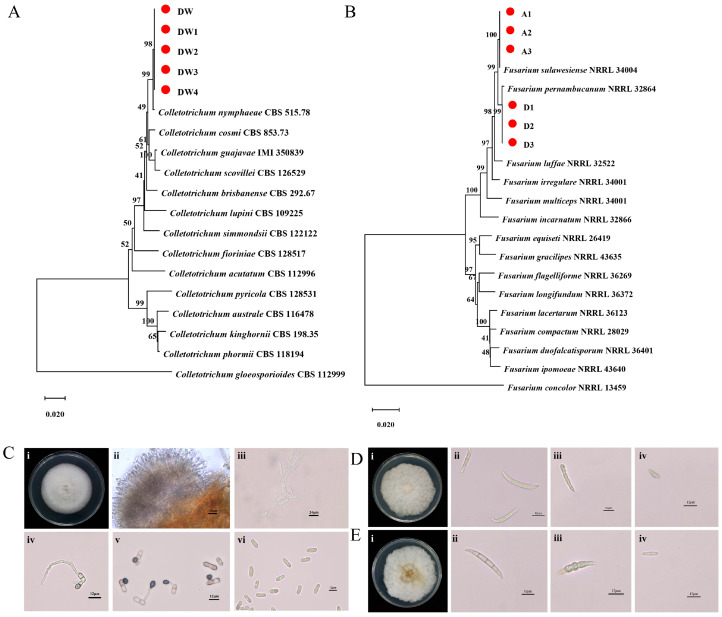
Identification of the strains isolated from plum brown spot disease. (**A**) Molecular status of strain DW based on multigene analysis of *GAPDH*, *Actin*, *TUB2* and *CHS-1* by maximum likelihood method of MEGA7.0 software. (**B**) Molecular status of strain (**A**,**D**) based on multigene analysis of *EF-1α*, *RPB2* and *CAM* by maximum likelihood method of MEGA7.0 software. (**C**) Morphological characteristics of strain DW. (**i**): Cultured for 7 days on PDA (**ii**): Acervulus (**iii**): Sporogenous structure (**iv**,**v**): Conidial appressorium (**vi**): Conidia (**D**) Morphological characteristics of strain A1. (**E**) Morphological characteristics of strain D2. (**i**): Cultured for 5 days on PDA (**ii**): Macroconidia (**iii**): Chlamydospores (**iv**): Microconidium.

To identify the pathogens responsible for the observed lesions, we isolated microorganisms from the diseased plum fruits, resulting in 11 distinct strains. Universal primers ITS1/ITS4 were employed to amplify the *ITS* region of these strains. Following sequencing and assembly, the sequences were compared using BLAST 2.17.0 in the GenBank database. Based on the comparison results, the strains were initially categorized into two groups: *Colletotrichum* spp. and *Fusarium* spp. For further intra-genus identification, specific primers were used. The results showed that 5 strains of *Colletotrichum* spp. clustered with *Colletotrichum nymphaeae*, supported by a bootstrap value of 98% (Figure 2A). These strains were designated as DW. The 6 strains of *Fusarium* spp. were divided into two subgroups (Figure 2B): 3 strains clustered with *Fusarium sulawesiense*, supported by a bootstrap value of 100%, and were designated as type A; the remaining 3 strains clustered with *Fusarium pernambucanum*, supported by a bootstrap value of 99%, and were designated as type D.

Subsequently, we carefully observed the morphological characteristics of the three types of strains. The results showed that strain DW was white on PDA medium, with pigmented colonies turning grayish white at later stages. The aerial hyphae were felt-like, tight, and flat, with neat edges and uniformly spread in concentric circles (Figure 2(Ci)). The vegetative hyphae were smooth-walled, septate, and unbranched (Figure 2(Civ)). The conidia were colorless, transparent, single-celled, straight cylindrical, without septa, blunt at both ends, with one end slightly narrower, generally containing an oil droplet, and measuring 13.775 ± 1.604 × 4.858 ± 0.536 μm (n = 100) (Figure 2(Cvi)). The spore attachment cells were single-celled, dark brown to black, smooth, round to oval, and measured 6.748 ± 0.810 × 5.831 ± 0.619 μm (n = 100) (Figure 2(Civ,Cv)). Conidial discs were scattered (Figure 2(Cii)), and the spore-producing structure of strain DW was observed as a spore stalk (Figure 2(Ciii)). The colony color of strain A1 on PDA was white to light yellow, with fluffy and flat aerial hyphae growing luxuriantly (Figure 2(Di)). The large conidia were transparent, slender, and typically sickle-shaped. The top cells were papillary, with 3 to 5 septa, measuring 28.002 ± 3.666 × 4.005 ± 1.550 μm (n = 100) (Figure 2(Dii)). The small conidia were oval, with 0 to 1 septa, measuring 11.069 ± 1.626 × 3.450 ± 0.290 μm (n = 100) (Figure 2(Div)). Both large and small conidia could form thick-walled spores (Figure 2(Diii)). In contrast, the colony color of strain D2 on PDA was white to light yellow, with fluffy and flat aerial hyphae growing vigorously, but sparse in the center of the colony at later stages (Figure 2(Ei)). The large conidia were transparent and sickle-shaped, with 3 to 5 septa, measuring 28.416 ± 5.241 × 5.241 ± 0.438 μm (n = 100) (Figure 2(Eii)). The small conidia were oval to elliptical, with 0 to 1 septa, measuring 9.855 ± 1.685 × 2.948 ± 0.329 μm (n = 100) (Figure 2(Eiv)). Both large and small conidia could produce thick-walled spores (Figure 2(Eiii)). These morphological characteristics are consistent with molecular identification, further substantiating the taxonomic status.

### 2.3. The Pathogenicity Testing of the Isolated Strains

**Figure 3:**
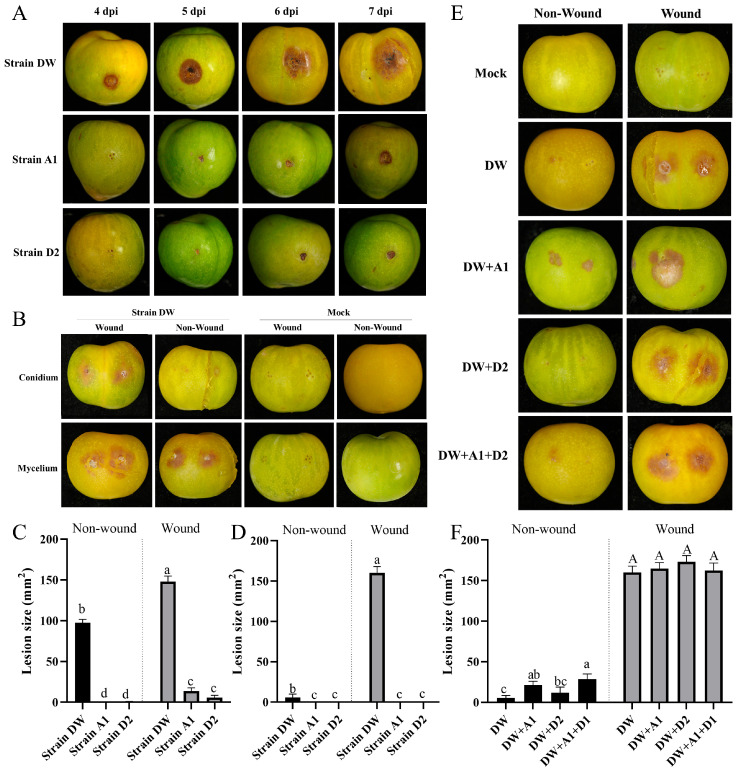
Pathogenicity test of isolated strains from plum brown spot disease. (**A**) Symptoms on plum fruits after inoculation of mycelium of three strains on different days with 28 °C 75%RH. (**B**) Effects of different inoculation methods on the pathogenicity of strain DW. (**C**) The lesions size at 6 dpi after the mycelium was inoculated on plum fruit by wounding/non-wounding method. (**D**) The lesions size at 6 dpi after the conidium was inoculated on plum fruit by wounding/non-wounding method. (**E**) Symptoms of plum fruit after combined infection with different strains by spore inoculation in wounding/non-wounding method. (**F**) Lesion size on plum fruit after combined infection with different strains. All experiments had been repeated three times with similar results, which every experiment with 9 samples. Values represent means ± standard error (SE) from three biological replications. The statistical analyses were performed using AVONA test (LSD, *p* < 0.05). Different letters in the figure indicates that the difference is significant (*p* < 0.05), identical letters indicates no significant difference (*p* > 0.05), Upper case and lower case letters correspond to two independent sets of comparison analyses, respectively.

To confirm that these isolated strains were the causative agents of plum brown spot disease, we conducted Koch’s postulates and pathogenicity tests. The mycelium block with wound inoculation method was employed for this purpose. Strain DW exhibited symptoms 2 days post-inoculation (dpi), with small plum brown spots appearing at the micro-wounds. By the third day, the lesions began to spread, forming brown circular spots. On the fourth day, the lesions continued to expand, displaying dark brown concentric circles at the outer edge, with light brown lesions spreading outward like water stains. By the fifth day, the lesions further expanded, becoming uniformly dark brown with neat edges. Between the sixth and seventh days, the lesions ceased significant expansion, and the affected fruit became severely rotten. The central lesion darkened to deep brown, featuring numerous small black dots (mycelium block adhesion) and a few yellow to orange dots (conidia disks) (Figure 3A). In contrast, strain A1 developed symptoms 4 dpi, presenting as small plum brown spots. By the fifth day, the lesions enlarged, and the center became noticeably sunken and hard (Figure 3A). Strain D2 also exhibited symptoms 4 dpi, initially showing small plun brown spots at the slight injury site. By the fifth day, the lesions expanded, further spreading and darkening in color over the next two days, with the lesions becoming sunken and hard (Figure 3A). The symptoms induced by these three strains on plum fruits were generally characterized by brown sunken spots, similar to those observed in the field-collected diseased fruits. After that, pathogens from the diseased areas were re-isolated and purified. Molecular biological identification was performed after DNA extraction, revealing that the gene fragment sequences of the re-isolated strains matched those of the original strains. This confirmed that strains DW, A1, and D2 were indeed the pathogens responsible for plum brown spot disease.

The above results demonstrate that all three isolated strains are pathogenic, indicating that plum brown spot disease in this region is a result of a complex infection. Consequently, we compared the pathogenicity differences among these strains. Considering the complex infection dynamics in the field, we employed both wound and non-wound inoculation methods to compare their pathogenicity. The results indicated that strain DW exhibited significantly higher pathogenicity compared to the other two strains under both wound inoculation and non-wound inoculation using mycelium pieces as the inoculum (Figure 3B). In contrast, strains A1 and D2 only displayed pathogenicity when inoculated with mycelium pieces, regardless of the inoculation method, and there was no significant difference in pathogenicity between them (Figure 3C). When spores were used as the inoculum, strains A1 and D2 showed no pathogenicity under either wound or non-wound inoculation conditions, whereas strain DW remained pathogenic (Figure 3B). Statistical analysis of lesion sizes revealed that strain DW is significantly more pathogenic to plum fruits than the other two strains (Figure 3D).

Finally, we compared the effects of polyinfections involving different strains on symptom production. Since compound infection requires spore inoculation, the spore infection from the combination of strains A1 and D2 did not show pathogenicity under both wounding and non-wounding inoculation methods. Therefore, we used strain DW as a background to determine whether compound spore infection exhibited enhanced pathogenicity. The results indicated that under the wound inoculation method, the pathogenicity of strain DW was not significantly different from the infections involving mixed strains A1, D2, or a combination of all three strains (Figure 3E). However, under the non-wound inoculation method, the pathogenicity of the combination strain DW + A1 and the combination strain DW + A1 + D2 was significantly stronger than the single infection of strain DW (Figure 3E). Specifically, the pathogenicity of the DW + A1 combination was more prominent, with darker lesions, while the lesion area of the DW + A1 + D2 combination was larger, measuring 28.67 ± 6.35 mm^2^ (Figure 3F). The pathogenicity of the combination strain DW + D2 did not significantly differ from that of single strain DW. Additionally, there was no significant difference in pathogenicity between the combination of strain DW + A1 and the combination of DW + D2, nor between the combination of strain DW + A1 + D2 (Figure 3F). This indicates that the pathogenicity of non-wound inoculation was significantly enhanced when strains A1 and D2 were mixed, but there was no significant effect on the pathogenicity of wound inoculation. These results suggest that the combination of strains DW and A1 is critical to the aggravation of the disease.

### 2.4. Growth Characteristics of the Pathogenic Strains

**Figure 4:**
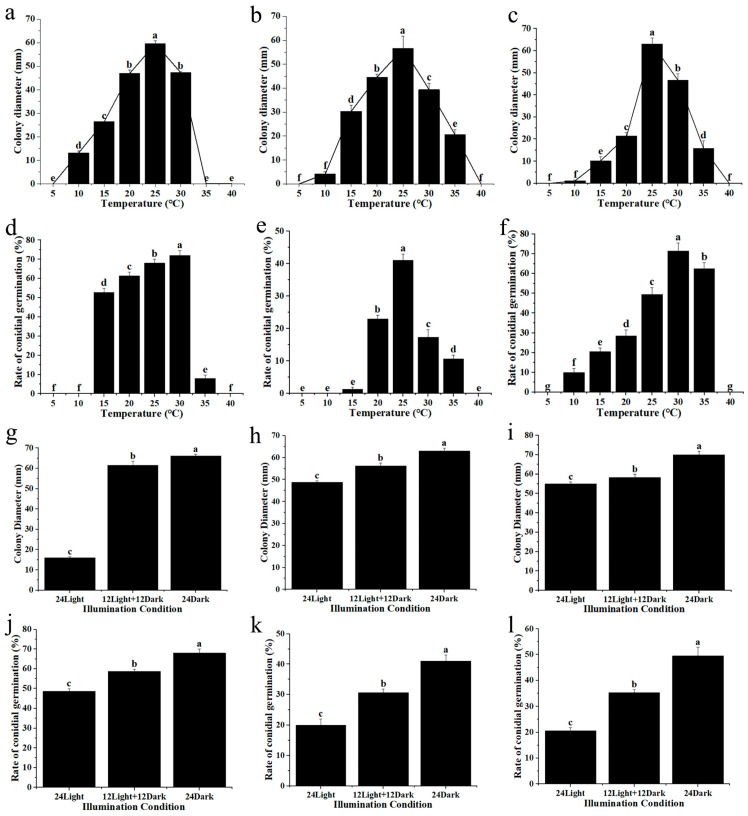
Adaptability of three strains to temperature and light. (**a**–**c**) Effects of different temperatures on the mycelial growth of strains DW, A1 and D2 in vitro. (**d**–**f**) Effects of different temperatures on the germination rate of spores of strains DW, A1 and D2. (**g**–**i**) Effects of different illumination times on the mycelial growth of strains DW, A1 and D2 in vitro. (**j**–**l**) Effects of different illumination times on the germination rate of spores of strains DW, A1 and D2. All experiments had been repeated three times with similar results. Values represent means ± standard error (SE) from three biological replications. The statistical analyses were performed using AVONA test (LSD, *p* < 0.05). Different letters in the figure indicates that the difference is significant (*p* < 0.05), identical letters indicates no significant difference (*p* > 0.05), Upper case and lower case letters correspond to two independent sets of comparison analyses, respectively.

Next, we conducted a detailed analysis of the growth characteristics of the three pathogens. Specifically, we examined their in vitro growth characteristics and spore germination rates at different temperatures. The results indicated that strain DW could grow within the temperature range of 10 °C to 30 °C. The overall growth trend of strain DW showed an initial increase followed by a decrease, with an optimum growth temperature of 25 °C (Figure 4a). The spore germination rate of strain DW was generally high, capable of germinating after 6 h of incubation within the temperature range of 15 °C to 35 °C. Between 15 °C and 30 °C, the spore germination rate exhibited a parabolic trend with increasing temperature, peaking at an optimal germination temperature of 30 °C (Figure 4d). Strain A1 could grow normally between 10 °C and 35 °C. The growth diameter of its mycelium increased with temperature up to 25 °C, which was identified as the optimal growth temperature (Figure 4b). The spore germination rate of strain A1 was generally low, with spores capable of germinating within the temperature range of 15 °C to 35 °C after 6 h. The overall trend of spore germination rate was parabolic, with an optimal germination temperature of 25 °C (Figure 4e). Strain D2’s mycelium could grow normally between 10 °C and 35 °C. The growth diameter of strain D2 showed a parabolic trend with temperature, with the optimal growth temperature being 25 °C (Figure 4c). The spores of strain D2 could germinate within the temperature range of 10 °C to 35 °C after 6 h. The spore germination rate also exhibited a parabolic trend, with the optimal germination temperature at 30 °C (Figure 4f). Understanding these optimal growth and spore production temperatures provides better insight into the primary pathogenic roles of these three strains in the region.

Afterwards, we investigated the influence of different lighting durations on the growth of the strains. The findings reveal that the hyphae and spores of strain DW exhibit normal growth and germination under conditions of 24 h of darkness, 12 h of darkness, 12 h of light, and 24 h of light. However, the growth diameter of the hyphae under complete darkness conditions is notably larger compared to those under light conditions (Figure 4g). Additionally, the spore germination rate is highest under total darkness conditions (Figure 4j), indicating that light significantly inhibits the mycelium growth and spore germination of strain DW. Similarly, the hyphae and spores of strains A1 and D2 demonstrate normal growth and germination under the three lighting conditions. Notably, lighting significantly inhibits the hyphae growth (Figure 4h,i) and spore germination (Figure 4k,l) of these strains.

### 2.5. BIOLOG Metabolic Characteristics of the Pathogenic Strains

**Figure 5:**
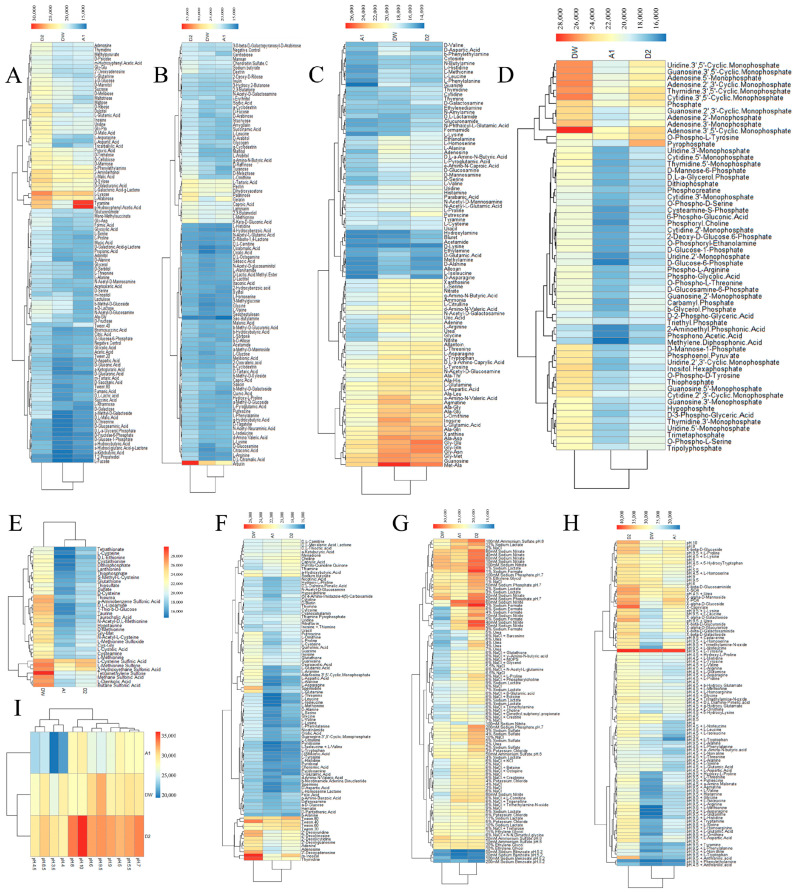
Metabolic utilization analysis of three strains based on BIOLOG phenotype microarray technology. (**A**,**B**) Carbon source utilization of three strains. (**C**,**D**) Nitrogen source utilization by three strains. (**E**) Utilization efficiency of sulfur sources by three strains. (**F**) Utilization efficiency of phosphorus sources by three strains. (**G**) Metabolism of three strains under different osmotic pressures and ionic strengths. (**H**,**I**) Metabolism of the three strains under different pH pathways.

To comprehensively elucidate the metabolic characteristics of the three strains, we employed BIOLOG Phenotype Microarray technology to analyze their metabolic profiles, encompassing various carbon, nitrogen, sulfur, and phosphorus sources, along with different stress conditions. The utilization rates of 190 carbon sources by strains DW, A1, and D2 were 44.21%, 40.00%, and 57.89%, respectively, with arbutin being the most utilized carbon source (Figure 5A,B). For 95 nitrogen sources, the utilization rates were 66.31%, 66.31%, and 68.42%, with ornithine, methionine–alanine, and glycine–methionine being the most utilized (Figure 5C). Regarding 94 biosynthetic pathway substances, the utilization rates were 11.70%, 50.00%, and 37.23%, respectively, with 2′ deoxynucleosides (AUCG muscle) being the most utilized (Figure 5D). The utilization rates of 59 phosphorus sources were 32.20%, 18.64%, and 22.03%, with nucleoside 3′,5′-cyclic phosphate (ATCGU) being the highest utilized (Figure 5F). The utilization rates of 35 sulfur sources were 54.29%, 71.43%, and 60.00%, with the most utilized being tetramethyl sulfoxide, L-cysteine sulfinic acid, and L-methionine sulfone (Figure 5E). The metabolic activity of the three strains varies under different osmotic pressures and ion strengths. Strain D2 demonstrated good metabolic activity under various osmotic pressures and ionic strengths. Sodium nitrate promoted the metabolism of all three strains, whereas sodium benzoate inhibited their metabolism (Figure 5G). The optimal pH for strain DW was 9.5, while strains A1 and D2 had an optimal pH of 10 (Figure 5I). The addition of amino acids under both acidic and alkaline conditions was not conducive to the metabolism of strains DW and D2. However, adding amino acids under acidic conditions slightly promoted the metabolism of strain A1. Additionally, the inclusion of L-tyrosine under alkaline conditions significantly enhanced the metabolism of all three strains (Figure 5H).

### 2.6. Pathogenic Characteristics of the Pathogenic Strains

**Figure 6:**
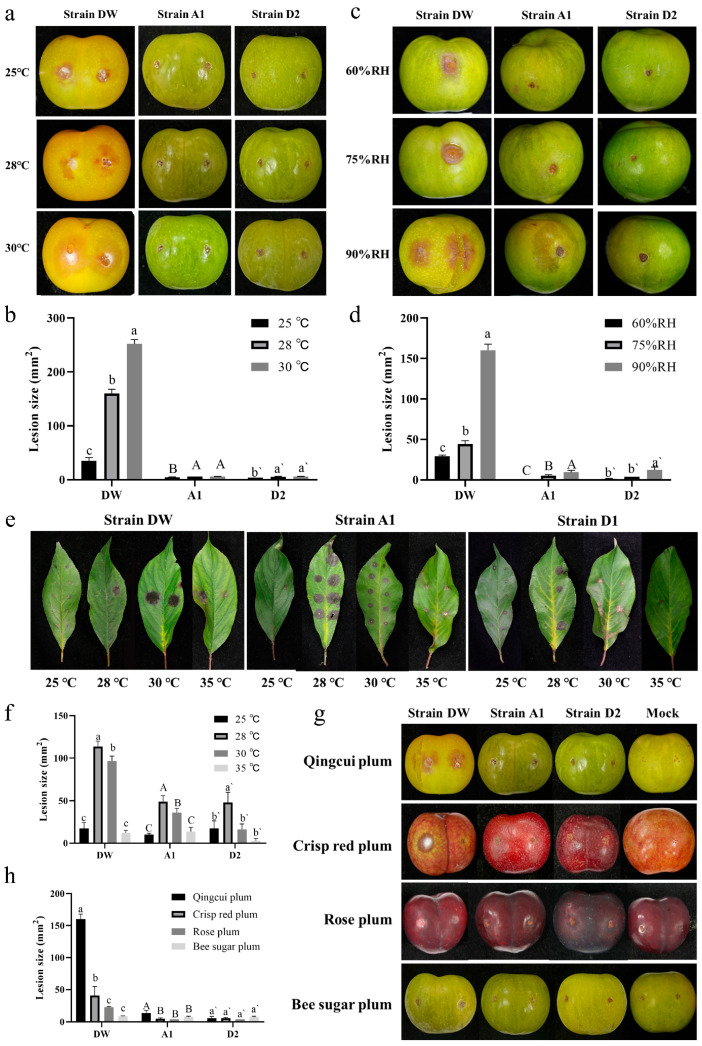
Pathogenicity characteristics of the three strains. (**a**,**b**) Comparison of pathogenicity of three strains at different temperatures. (**c**,**d**) at 7 dpi. Comparison of pathogenicity of three strains under different humidity at 5 dpi. (**e**,**f**) Comparison of pathogenicity of three strains on plum tree leaves at 7 dpi. (**g**,**h**) Differences in pathogenicity of three strains on different plum varieties at 7dpi. All experiments had been repeated three times with similar results. Values represent means ± standard error (SE) from three biological replications. The statistical analyses were performed using AVONA test (LSD, *p* < 0.05). Different letters in the figure indicates that the difference is significant (*p* < 0.05), identical letters indicates no significant difference (*p* > 0.05), Upper case and lower case letters correspond to two independent sets of comparison analyses, respectively.

To formulate effective prevention and control strategies, we conducted a detailed study of the pathogenic characteristics of the three strains. First, we analyzed the effect of temperature on their pathogenicity. Our findings revealed that strain DW exhibited significant variability in pathogenicity across three temperature settings (25, 28 and 30 °C), with lesion size increasing as temperature rose (Figure 6a). At 30 °C, the lesion area reached 252.00 ± 8.00 mm^2^. Since fruit rots if cultivated above 30 °C for seven days, we determined that 30 °C is the optimal temperature for strain DW to infect fruits. For strains A1 and D2, there was no significant difference in pathogenicity between 28 °C and 30 °C, but both temperatures resulted in significantly larger lesion areas compared to 25 °C, indicating an optimal infection temperature range of 28–30 °C (Figure 6b). Additionally, we inoculated the strains with mycelium pieces and cultured them for five days under three different humidity conditions (60%, 75% and 90%). All three strains demonstrated pathogenicity under each condition, with significant differences in lesion sizes. The largest lesions occurred at 90% humidity, while the smallest were at 60%, indicating that high humidity significantly enhances the pathogenicity of these strains (Figure 6c,d). These results suggest that the pathogens responsible for plum brown spot thrive under high temperature and high humidity conditions. This understanding is crucial for developing targeted strategies to manage and control the spread of the disease effectively.

Subsequently, we analyzed whether the three pathogenic strains can infect plum tree leaves. Notably, all three strains exhibited pathogenicity towards plum leaves at various temperatures. The infection symptoms caused by strain DW were characterized by black concentric circular spots. In contrast, the symptoms induced by strains A1 and D2 were similar but often included small, hard white dots in the center. Specifically, strain DW exhibited the highest pathogenicity at 28 °C, with a lesion area of 113.67 ± 6.35 mm^2^, which was significantly larger than at other temperatures. At 30 °C, the lesion area was 96.67 ± 5.77 mm^2^. At 25 °C and 35 °C, there was no significant difference in pathogenicity, indicating that the optimal temperature for strain DW to infect leaves is 28 °C. The lesion area of strain A1 at 28 °C was 49.00 ± 7.00 mm^2^, significantly larger than at other temperatures, establishing 28 °C as the optimal leaf infection temperature. The pathogenicity was next highest at 30 °C, with no significant difference between 35 °C and 25 °C. For strain D2, the optimal leaf infection temperature was 28 °C, with a lesion area of 48.00 ± 12.00 mm^2^, significantly larger than at other temperatures. No significant differences in pathogenicity were observed at 25 °C, 30 °C, and 35 °C (Figure 6e,f).

After that, we compared the pathogenicity of the strains on plum fruits and leaves at different temperatures to analyze the differences in their pathogenicity towards different parts of plums. The results indicated significant differences in the pathogenicity of strain DW on different parts of plums. The optimal infection temperature for fruits was 30 °C, while for leaves, it was 28 °C. Additionally, the pathogenicity of strain DW on fruits was significantly stronger than on leaves at all three temperatures, indicating a higher overall pathogenicity towards plum fruits. In contrast, the pathogenicity of strain A1 on leaves was significantly stronger than on fruits at all three temperatures. The largest lesion area on fruits was smaller than the smallest lesion area on leaves, indicating that strain A1 is more pathogenic to plum leaves. For strain A1, the optimal temperature for infecting fruits was 30 °C, while for leaves, it was 28 °C. Strain D2 also showed differences in pathogenicity towards different parts of plums. The maximum lesion area on fruits occurred at 30 °C, while on leaves, it was at 28 °C. Strain D2 exhibited significantly stronger pathogenicity towards leaves than fruits at 25 °C (0.001 < *p* < 0.01) and 28 °C (*p* < 0.001), with no significant difference at 30 °C (Figure S1). Thus, strain D2 generally displayed stronger pathogenicity when infecting plum leaves. Interestingly, the differential pathogenicity in various parts may enable the three pathogenic strains to form growth cycles in different parts, facilitating their long-term survival.

Finally, we selected four plum varieties—Qingcui plum, rose plum, crisp red plum, and bee sugar plum—that are widely sold in the market to evaluate the pathogenicity differences of the three strains on their fruits. The results indicated that strain DW exhibited the highest pathogenicity towards Qingcui plum among the four varieties. It was followed by crisp red plum, with a lesion area of 41.00 ± 13.86 mm^2^. For rose plum and bee sugar plum, the pathogenicity was significantly lower and showed no significant difference between the two varieties. Strain A1 also demonstrated significantly stronger pathogenicity towards Qingcui plum compared to other varieties, with no significant differences observed in pathogenicity among the other varieties. Conversely, strain D2 caused similar lesion areas across all four plum varieties, indicating no significant difference in pathogenicity among them (Figure 6g,h). These findings suggest that Qingcui plum has the lowest resistance to the three pathogenic strains, while rose plum and bee sugar plum exhibit relatively strong resistance.

### 2.7. Screening of Chemical Agents for the Management of Plum Brown Spot

The foundation of pathogen identification is prevention and control. To screen chemical agents that effectively prevent and treat the three pathogens, we evaluated 16 chemical agents with different modes of action. The results showed that, after preliminary screening, seven fungicides were effective against strain DW, including tolclofos-methyl, prochoraz-manganese, difenoconazole, pyraclostrobin, fluazinam, fludioxonil, and mancozeb. Among these, prochoraz-manganese and fluazinam demonstrated the best antibacterial effects, achieving an antibacterial rate of 100.00%. For strain A1, eight fungicides were identified: tolclofos-methyl, prochoraz-manganese, trifloxystrobin, triadimefon, pyraclostrobin, fluazinam, fludioxonil, and hymexazol. Of these, the imidazole family fungicides prochoraz-manganese andtrifloxystrobin had an inhibitory rate of 100.00% against strain A1. For strain D2, fourfungicides were effective: fluazinam, trifloxystrobin, tolclofos-methyl, prochoraz-manganese and fludioxonil. Fluazinam, a dinitrogen aniline fungicide that inhibits respiration, had the best antifungal effect, with an antifungal rate of 87.20% (Table S7). Subsequently, we analyzed the effects of these chemicals on mycelium growth and spore germination of the three pathogenic strains. We examined their virulence regression equations, correlation coefficients, *EC50* values, and 95% confidence intervals. prochoraz-manganese had the most potent inhibitory effent on the mycelial growth of strain DW, with an *EC50* of 0.0686 μg/mL. Fludioxonil, fluazinam, pyraclostrob in, and difenoconazole showed strong inhibition, with *EC50* values ranging between 0.1000 μg/mL and 0.9000 μg/mL (Table S8). The best inhibitor of spore germination for strain DW was pyraclostrobin, with an *EC50* of 0.0018 μg/mL. Fluazinamdifenoconazole and prochoraz-manganesealso strongly inhibited spore germination, with *EC50* values below 1.000 μg/mL (Table S9). For strain A1, fluazinam was the most effective against mycelial growth, with an *EC50* of 0.2970 μg/mL, followed by trifloxystrobin, with an *EC50* of 0.8283 μg/mL (Table S10). Trifloxystrobin was the most effective inhibitor of spore germination, with an *EC50* of 0.0321 μg/mL. Fludioxonil, pyraclostrobin fluazinam and prochoraz-manganesealso showed strong inhibition, with *EC50* values below 1.000 μg/mL (Table S11). For strain D2, fluazinam had the best inhibitory effect on mycelial growth, with an *EC50* of 0.4447 μg/mL. Trifloxystrobin and fludioxonil also had strong inhibitory effects, with *EC50* values below 1.000 μg/mL (Table S12). Fluazinam was the most effective against spore germination, with an *EC50* of 0.0049 μg/mL. Fludioxonil, prochoraz-manganese and trifloxystrobin also strongly inhibited spore germination, with *EC50* values below 1.000 μg/mL (Table S13).

Since strain DW is the primary agent of plum brown spot disease, we further investigated the in vivo control efficacy of the most promising chemical agents. Specifically, we evaluated both the protective and therapeutic effects of these agents, measuring their control effectiveness on plum brown spot disease before and after pathogen inoculation. The results indicated that for the protective effect, the control efficacy of 50% fluazinam SC on detached fruits infected with strain DW at concentrations ranging from 25 μg/mL to 80 μg/mL was above 50.00%. At the concentration of 80 μg/mL, the protective effect reached 96.06%. Similarly, 45% prochloraz–manganese EW demonstrated a control efficacy above 50.00% at concentrations of 10 μg/mL and higher, with a control effect of 94.74% at 50 μg/mL.Furthermore, 30% pyraclostrobin SC showed a control efficacy above 50.00% at concentrations from 1 μg/mL to 10 μg/mL, achieving 100.00% at 10 μg/mL. Regarding the therapeutic effect, the efficacy of 50% fluazinam SC on in vitro fruits infected with strain DW at concentrations from 25 μg/mL to 80 μg/mL was also above 50.00%, with a control effect of 91.35% at 80 μg/mL. For 45% prochloraz–manganese EW, the control efficacy at 25 μg/mL and higher was above 50.00%, reaching 94.54% at 50 μg/mL. The therapeutic control efficacy of 30% pyraclostrobin SC at concentrations ranging from 1 μg/mL to 10 μg/mL was above 50.00%, achieving 100.00% at 10 μg/mL (Table 1). These findings provide a theoretical foundation for the effective prevention and control of plum brown spot disease, supporting the use of specific chemical agents to manage this significant agricultural issue.

## 3. Discussion

Three fungal strains, *Colletotrichum nymphaeae*, *Fusarium sulawesiense* and *Fusarium pernambucanum*, were isolated and identified from the lesions of Qingcui plum in Chongqing in this study. Given that the lesions are dark brown and that strains belonging not only to the genus *Colletotrichum* were isolated from them, this disease was named plum brown spot.

*C. nymphaeae*, part of the *C. acutatum* species complex, is a significant group within the genus *Colletotrichum*, consisting of 41 species infecting 171 species across 129 genera, predominantly dicots (90.9%), with fewer monocots (5.3%) and gymnosperms (1.6%) [22]. This complex typically features transparent, colorless conidia with smooth walls, straight cylindrical shapes, and sharp or thin ends. The conidiophores are orange or pink, with bristles rarely observed in culture or host environments [23]. The morphological characteristics of strain DW in this study align with these descriptions. Although *C. nymphaeae* has been reported on Japanese plum fruits in South Korea [19], only molecular identification was conducted without further morphological and pathogenic studies, and there are no records of this pathogen affecting plum fruits in China. Hence, *C. nymphaeae* represents a new pathogen for Chinese plum fruits. *F. sulawesiense* and *F. pernambucanum* belong to the *F. incarnatum*-equiseti species complex (FIESC), a diverse group associated with various crop diseases and containing over 30 cryptic species, complicating phenotypic identification [24]. Molecular identification using the *ITS* sequence, historically significant in *Fusarium* research, has limitations for FIESC species. In contrast, *EF-1α* sequence analysis offers higher resolution and can distinguish strains within FIESC into branches such as *F. incarnatum* and *F. equiseti* [25]. Additionally, the CAM gene identifies *F. semitectum* within FIESC, and the *RPB2* gene effectively identifies *F. sulawesiense*, *F. hainanense*, *F. bubalinum*, and *F. tanahbumbuense* [26]. Therefore, this study employed EF-1α, RPB, and CAM genes for phylogenetic analysis. For more accurate molecular identification, strain ID numbers preserved by the United States National Reserve Library (NRRL) were used to construct strains A1 and D2, with *F. concolor* as the outgroup. The morphological characteristics of large and small conidia from these strains matched those of *F. sulawesiense* and *F. pernambucanum* isolated from Sichuan plum leaf blight [27]. These fungi are known to infect fruits, causing rot, as seen with Brazilian melon rot [28].

The optimal mycelial growth temperature of the three strains was determined to be approximately 25 °C, while the optimal temperature range for spore germination was 25–30 °C. Light exposure was found to exert a significant inhibitory effect on both mycelial growth and spore germination of the strains. Based on these findings, it can be inferred that the most favorable geographical conditions for the outbreak and prevalence of this disease are regions characterized by low light intensity and temperatures ranging from 25 to 30 °C. Subsequently, Biolog phenotypic microarrays were employed to investigate the phenotypic characteristics of the three strains, leading to the identification of several substances with considerable research value. Arbutin, chemically known as 4-hydroxyphenyl-β-d-glucopyranoside, is predominantly found in frost-resistant plants such as pear [29] and Rhodiola rosea [30]. It acts as a tyrosinase inhibitor, competing to block the synthesis of dopa and dopaquinone, thereby inhibiting melanin synthesis [31]. In this experiment, arbutin significantly promoted the metabolism of pathogenic strains as a carbon source. However, academic research on arbutin primarily focuses on its synthesis, extraction, and application. Further research is needed to elucidate the relationship between arbutin and the metabolism of pathogenic strains. Phosphorus is crucial for nucleic acids, proteins, phospholipids, and plays an important role in glycolysis, respiration, enzyme activity, and energy storage and transfer [32]. In this experiment, strain DW, which showed the highest pathogenicity, also exhibited the highest utilization type and ratio of phosphorus sources. All three strains utilized nucleoside 3′,5′-cyclic phosphate (ATCGU) at a high rate, particularly adenosine 3′,5′-cyclic phosphate (cAMP). The cAMP signaling pathway is closely associated with pathogenic strain’s ability to sense host surface signals, appressorium formation, and pathogenic processes [33]. External application of cAMP has been shown to increase extracellular cellulase synthesis levels in filamentous fungi [34], which are crucial cell wall-degrading enzymes for fungal infection and colonization [35]. Therefore, the high rate of cAMP utilization by more pathogenic strains suggests a potential link between cAMP utilization and pathogenicity in filamentous fungi. In addition, the metabolic conditions of the three strains varied under different osmotic pressures and ionic strengths. Strain D2 not only metabolized well under most stresses but also exhibited the highest overall metabolic level, indicating strong stress resistance. However, despite this, strain D2 did not produce the largest lesion area when infecting the host, suggesting that stress resistance under different osmotic pressures and ionic strengths may not directly correlate with pathogenicity. Sodium benzoate significantly inhibited the metabolism of all three strains. As a highly lipophilic broad-spectrum food preservative, sodium benzoate can penetrate cell membranes, disturb permeability, inhibit amino acid absorption, acidify intracellular reserves, inhibit respiratory enzyme activity, and block acetyl-CoA condensation [36]. It is hypothesized that sodium benzoate inhibits the metabolic activity of the three strains through these mechanisms, suggesting potential applications in production practices, though further experimental verification is required. Adding L-tyrosine under alkaline conditions significantly promoted strain metabolism. L-tyrosine can generate eumelanin through tyrosine ammonia lyase (TAL) and p-coumaric acid-3-hydroxylase (C3H), producing melanin based on protocatechualdehyde [37]. Melanin helps fungi resist environmental stress and is related to the pathogenicity of many fungi, such as *Aspergillus fumigatus* [38]. In *Colletotrichum*, melanin is linked to the integrity of the appressorium and the accumulation of internal pressure for host penetration [39]. Since L-tyrosine is easily soluble in alkali, it is speculated that adding L-tyrosine under alkaline conditions enhances stress resistance by synthesizing melanin, thereby promoting strain metabolism. However, studies have shown that L-tyrosine can promote pyomelanin production in *Alternaria* fungi, inhibit DHN-melanin synthesis, and reduce the chitin content and thickness in fungal cell walls, without affecting infection and colonization [40]. Therefore, the impact of L-tyrosine varies among strains, necessitating further research to understand its mechanisms.

The infection process of pathogenic bacteria on host plants consists of contact, penetration, incubation, and symptom development, among which penetration is the prerequisite for pathogenic fungi to spread within the host and induce diseases subsequently. *Colletotrichum* and *Fusarium* species employ three penetration modes: direct penetration, natural opening penetration, and wound penetration. Direct penetration requires the pathogen to generate specialized infection structures after contacting the host surface, followed by enzymatic degradation and mechanical force to breach the host cuticle. In contrast, natural opening penetration and wound penetration do not require the formation of such infection structures [41]. Specifically, wound penetration enables pathogens to rapidly invade and colonize the host tissues, and the mechanical damage created by wounds also facilitates the pathogen’s penetration through the host cuticle. This finding aligns with the stronger infectivity of pathogens observed in the wound inoculation treatment of this study. Notably, the two *Fusarium* isolates in this study exhibited pathogenicity on plum fruits only at 30 °C under the unwounded inoculation condition. This further indicates that these two *Fusarium* isolates have weak active infectivity toward plum fruits, suggesting that they may act as opportunistic pathogens. *Fusarium* species typically infect the roots or leaves of plants. Lu et al. [27] isolated three *Fusarium* species—*F. citri*, *F. sulawesiense*, and *F. pernambucanum*—from the diseased tissues of plum trees exhibiting leaf blight. Among these isolates, the latter two species are the same *Fusarium* strains obtained from plum fruits in the present study. The symptoms caused by these pathogens were characterized by chlorotic small spots at the initial stage, which subsequently developed into grayish-brown–dark brown lesions. These symptoms were consistent with those observed on plum leaves inoculated with the two *Fusarium* strains in this study. Infection of fruits by *Fusarium* species is relatively rare, but there are documented cases such as muskmelon fruit rot [42] and watermelon fruit rot caused by *F. equiseti* [28]. Based on the comprehensive analysis of the following findings: (1) the two *Fusarium* isolates caused only mild damage to plum fruits in this study; (2) these two species have been reported as pathogens of plum leaves, with more severe pathogenicity on leaves than on fruits; and (3) *Colletotrichum* species were identified as the primary pathogen responsible for the plum fruit disease in this study, it is hypothesized that the two *Fusarium* strains may infect plum fruits secondarily through contact after the fruits have been initially infected and damaged by *Colletotrichum* species. However, the specific infection process requires further investigation. On this basis, the present study selected *Colletotrichum* species as the primary pathogenic fungi to further investigate the effects of different strain combinations on the infection of plum fruits. The results showed that in the wound inoculation treatment, the lesion area caused by the mixed inoculation of *Colletotrichum* with the two *Fusarium* isolates showed no significant difference compared with that caused by the single inoculation of *Colletotrichum*. By contrast, in the unwounded inoculation treatment, the lesion area induced by the mixed inoculation was significantly enlarged. These findings demonstrate that the *Fusarium* isolates can facilitate the infection of plum fruits by *Colletotrichum* species, thereby exacerbating the disease damage. Regarding such mixed infection, existing literature has documented that *Fusarium* pathogens are capable of co-infecting host plants together with other pathogenic microorganisms, thereby exacerbating the disease damage to the host. For instance, the co-infection of *Glycine max* (soybean) by *Fusarium solani* and *Heterodera glycines* (soybean cyst nematode) increases the incidence of sudden death syndrome [43]. Similarly, the combined infection of *Ipomoea batatas* (sweet potato) by *Alternaria tenuissima* and *Fusarium oxysporum* induces stem blight, and the pathogenicity of this mixed infection is significantly stronger than that of infection by either pathogen alone [44]. Researchers from Nanjing Agricultural University have reported that *Phytophthora sojae* and *Fusarium* species frequently coexist in the diseased root tissues of soybeans affected by root rot. Furthermore, *Fusarium* can enhance the pathogenicity of *P. sojae* by secreting vitamin B6 to suppress the expression of disease-resistant genes in soybeans [45]. Mixed infections involving *Fusarium* and *Colletotrichum* species have also been reported in previous studies. For example, Li et al. [46] isolated and identified the causal agents of soybean anthracnose, and confirmed the occurrence of mixed infections by *Colletotrichum truncatum*, *Fusarium proliferatum*, and *Fusarium equiseti*. Based on pathogenicity assays, *C. truncatum* was identified as the primary pathogen responsible for this disease. However, the facilitative mechanisms underlying the mixed infection of *Colletotrichum* and *Fusarium* species have not been reported to date and thus warrant further investigation.

The study further investigated the effects of environmental factors on the infection of plum hosts by the strains. The optimal temperature for the three strains to infect plum fruits was found to be 30 °C, while the optimal temperature for infecting plum leaves was 28 °C. High humidity and a lack of light significantly promoted the damage caused by all three strains to plum fruits. Additionally, the three strains demonstrated pathogenicity towards the fruits of four different plum varieties, with the strongest pathogenicity observed in Qingcui plum. Thus, environmental conditions characterized by high temperature, high humidity, and darkness are the most conducive for the infection and damage caused by plum brown spot disease. The Qingcui plum is a mid-ripening variety, with its maturity period occurring in early July. Consequently, it is speculated that the infection, damage, and outbreak of the disease occur from the maturity to the harvest period of Qingcui plum. During this period, it is crucial to focus on ventilation and heat dissipation, pruning branches, ensuring sufficient light, and taking preventive measures before the rainy season.

Chemical control is known for its rapid action, effective results, and ease of use. Despite its disadvantages, such as environmental pollution and the potential for developing resistance, it remains the most commonly used method for plant disease management and is the primary means for preventing and controlling new diseases. A total of 13 tested test chemical agents were screened in this study. Based on their inhibitory effects on the mycelial growth and spore germination of the target strains, 98% fluazinam technical concentrate (TC) exhibited excellent inhibitory activity against strains A1 and D2. In contrast, strains DW showed high sensitivity to three fungicides, namely 96%, prochloraz–manganese chloride complex TC, 98% pyraclostrobin TC, and 98% fluazinam TC. Given that strain DW was identified as the primary pathogen responsible for plum brown spot, the formulated preparations of these three fungicides were selected for subsequent in vitro fruit bioassays. Among them, the 30% pyraclostrobin suspension concentrate (SC) achieved the highest protective and curative efficacies against DW infection in detached plum fruits. Prochloraz–manganese, a combination of prochloraz and manganese chloride, is a broad-spectrum, high-efficiency, low-toxicity imidazole fungicide that acts as a sterol demethylation inhibitor (DMI). Its strong protective effect makes it more suitable as a preventive agent in field applications. Pyraclostrobin, a methoxyacrylate fungicide with a pyrazole structure, has demonstrated good control effects on various plant fungal diseases [47]. However, prolonged use has led to resistance in some pathogens, such as *C. siamense* [48]. Pyraclostrobin’s high compatibility allows it to be used in combination with other fungicides to reduce resistance. Fluazinam, a pyridinamine derivative, is a highly efficient, broad-spectrum, and long-lasting protective fungicide with low resistance risk and minimal cross-resistance with other fungicides [49]. However, improper use can cause crop damage, making it advisable to combine fluazinam with other fungicides to maximize its benefits and minimize risks [50]. Given fluazinam’s high sensitivity to strains A1 and D2 in the indoor toxicity test, it is recommended to use a combination of fluazinam and pyraclostrobin, or a combination of all three agents, for disease control in the field. However, this study did not include combination tests, and the optimal scientific ratio between fungicides requires further testing. All tests were conducted indoors, without accounting for varying field conditions such as temperature, humidity, light, and soil environments. Therefore, this study’s findings need to be validated through field efficacy tests.

This study investigated the pathogen identification, infection characteristics, and control strategies of plum brown spot, thereby providing a theoretical basis for the prediction and management of this disease. However, several limitations remain in the present study, and further in-depth investigations are required regarding the following aspects: (1) Field surveys and sample collections were conducted on plum brown spot occurring in plum orchards across different regions, so as to clarify the disease incidence, onset time, and dominant pathogenic fungi of plum brown spot in various regions. (2) Further exploration of the Biolog assay data, combined with the determination of corresponding metabolite contents in plum fruits at different stages post-pathogen inoculation via high-performance liquid chromatography (HPLC). This will clarify the utilization patterns of various substances by the pathogens in the actual host tissues. (3) Comparative analysis of the results obtained from the HPLC-based metabolite detection (as described in 2) and the Biolog assay. Focus should be placed on the substances that were significantly utilized by all three strains in both assays, including analyses of the molecular mechanisms underlying their biosynthesis pathways and their biological functions. These analyses should be further correlated with pathogenicity characterization to elucidate the pathogenic mechanisms of the target pathogens. (4) Laboratory-based compounding tests of the screened fungicides. A scientific formulation with optimal disease control efficacy should be developed by comprehensively considering fungicide resistance risks and environmental impacts. Subsequently, the compound fungicide with the highest toxicity against the pathogens should be selected for in vitro fungicide efficacy tests using detached plum fruits. (5) Field efficacy trials to validate the performance of the screened fungicides, aiming to provide more reliable data and optimal application rates for practical agricultural production. (6) Pesticide residue analysis during the field trials to evaluate the safety of the applied fungicides to the host plants.

## 4. Materials and Methods

### 4.1. Sample Collection and Pathogen Purification

During a fruit disease investigation conducted in Wanzhou, Chongqing in 2021 (30°44′38.30″ N, 108°5′14.43″ E), diseased plum fruits exhibiting reddish-brown–dark brown sunken round lesions were discovered. The lesion area ranges from 9.00 mm^2^ to 150.00 mm^2^. As a pre-harvest disease occurring during the fruit-ripening stage, it has affected an area of 2533.33 hectares, leading to a yield loss of 33,000 metric tons. To ascertain the underlying cause of this phenomenon, the affected and healthy parts of plum fruits were meticulously dissected into quadrilaterals approximately 4 mm^2^ in size. These tissue samples were then subjected to a series of surface sterilization procedures. Initially, they were immersed in 75% alcohol for 30 s, followed by a 2 min treatment with a 5% sodium hypochlorite solution, and finally rinsed thrice with sterile water. After air-drying on filter paper, the tissue fragments were placed onto potato dextrose agar (PDA) culture medium and incubated in darkness at a constant temperature of 25 °C for a duration of 5 days. Subsequently, sterile water was added to the surface of the culture medium, and a sterilized brush was used to disperse the hyphae and spores present. The resulting mixture was evenly spread, and a droplet was transferred onto a glass slide for microscopic observation. Once conidia were clearly visible, a 200 μL aliquot of the mixed solution was carefully deposited onto water agar (WA) culture medium using a dropper. After spreading evenly with a spreader, the culture dish was inverted under a microscope. Upon identifying a single spore within the field of view, the corresponding position was marked, and a section of the WA medium at that location was excised. This section was then placed upside down onto fresh PDA medium and incubated in darkness at 25 °C. The purified strain obtained from this process was preserved in glycerol at −80 °C for subsequent use.

### 4.2. Pathogen Identification

Following the aforementioned separation and purification procedures, a total of 11 single-spore strains were successfully isolated. Initially, universal sequence intra ribosomal transcribed spacer (*ITS*) identification was conducted on these 11 isolated single spores. Specifically, DNA extraction from the strains was performed utilizing a DNA extraction kit (Guangzhou Magen Biotech Co., Ltd., D3171-02, Guangzhou, China). Subsequently, amplification was carried out using 2× Es Taq MasterMix (Jiangsu ComWin Biotech Co., Ltd., CW0690H, Jiangsu Taizhou, China), followed by ligation into pGEM^®^-T Easy Vector Systems (Promega (Beijing) Biotech Co., Ltd., A137A, Beijing, China) and submission for sequencing at Sangon Biotech (Sangon, Shanghai, China). Based on the sequencing results, the strains were categorized into three distinct types, denoted as DW, A, and D, respectively. Subsequent to the initial classification, specific primers corresponding to the genus identification of the three types of strains were employed. For the DW strain, glyceraldehyde-3-phosphate dehydrogenase (*GAPDH*), actin (*ACT*), β-tubulin gene 2 (*TUB2*), and chitin synthase A (*CHS-1*) genes were targeted for amplification, while the translation elongation factor1-alpha (*EF-1α*), the second largest RNA polymerase subunit (*RPB2*), and calmodulin (*CAM*) genes of the A and D strains were amplified. Primer sequences were synthesized by Shanghai Sangon Biotechnology Co., Ltd., Shanghai, China. (refer to Table S1 for primer information). The PCR reaction system comprised 10 μL of 2× Es Taq MasterMix, 1 μL of forward primer, 1 μL of reverse primer, 1 μL of extracted DNA, and 7 μL of ddH_2_O, totaling 20 μL. The amplification program entailed an initial denaturation step at 95 °C for 5 min, followed by 32 denaturation cycles comprising denaturation at 95 °C for 30 s, annealing for 30 s (specific annealing temperatures provided in Table S1), extension at 72 °C for 45 s, and a final extension at 72 °C for 10 min. All PCR reactions were executed utilizing Biometra TOne 96 G (Analytik Jena Thermocycler, Jena, Germany). Subsequently, the amplified products underwent bidirectional sequencing at Shanghai Sangon Biotechnology Co., Ltd.

Following sequencing, bidirectional sequence fragments were concatenated into single sequences utilizing SeqMan software 7.1.0 (Purchased uniformly by the laboratory). Subsequently, PhyloSuite software v1.2.2 facilitated MAFFT merging, alignment, trimming, and concatenation of sequences from the test strains alongside sequences from each authoritative strains for evolutionary tree construction. The resultant processed sequences were saved in fasta format and imported into MEGA 10.2.6 software. Utilizing the maximum likelihood (ML) tree was constructed, with the bootstrap value set at 1000 iterations.

Finally, morphological identification was conducted by culturing the purified strains on PDA plates in darkness at 25 °C for a duration of one week. Following this incubation period, observations were made regarding colony color, morphology, and texture (*n* ≥ 50). Microscopic examination was performed using sterile water as the mounting medium using the BK series biological microscope (Nikon Precision Machine (Shanghai) Co., Ltd., Eclipse E200, Shanghai, China).

### 4.3. Pathogenicity Test

To verify Koch’s postulates, representative strains from each of the three isolated strains were selected, and their pathogenicity was assessed through mycelial block inoculation and spore inoculation methods. For mycelial block inoculation, mycelial blocks grown on PDA medium for one week (diameter = 5 mm) were utilized. The mycelial side was inoculated onto the surface of plum fruits, with separate groups designated for wounded and non-wounded inoculations. Controls were inoculated with PDA medium alone. Spore suspension inoculation involved preparing 1 × 10^5^ spores/mL suspension for each of the three strains. Subsequently, 20 μL of the respective spore suspension was applied to the surface of plum fruits, both wounded and unwounded. After 7 days, the lesion sizes were measured. Furthermore, to investigate mixed invasion, spore suspensions of the strains were mixed in a 1:1:1 ratio and applied in the same manner as described above. After incubation in a dark, humid environment at 28 °C for one week, the lesion areas were measured. Plum fruits inoculated with 20 μL of sterile water served as controls for comparison.

Furthermore, we evaluated the pathogenicity of these strains on plum leaves. The strains were inoculated onto the surface of plum leaves using the mycelial block wound inoculation method and then placed in incubators set to 25 °C, 28 °C, 30 °C, and 35 °C for 7 days. Lesion sizes were measured using the cross method. Uninoculated PDA culture medium served as a blank control, and each treatment was performed in triplicate.

We also analyzed the pathogenicity differences of the three types of strains under various temperatures and humidity levels. Following inoculation using the mycelial block method, the fruits were incubated in the dark at 25 °C, 28 °C, and 30 °C for 7 days to assess lesion size. PDA medium without mycelial block served as a blank control, and each treatment was performed in triplicate. To evaluate the impact of humidity, inoculated fruits were incubated at 28 °C with relative humidity levels of 60%, 75%, and 90%. Lesion diameters were measured after 5 days. (Fruits inoculated with the target strain completely rotted 7 days post-inoculation under 95% relative humidity.)

Finally, we evaluated the differences in pathogenicity among various plum cultivars. Fresh Qingcui plums, Crisp red plums, Rose plums, and Fengtang plums were selected and inoculated using the mycelial block wound inoculation method. The inoculated fruits were then placed in a 28 °C incubator for 7 days in the dark. Lesion sizes were measured using the cross method to determine the pathogenicity differences among the cultivars.

### 4.4. Growth Characteristics of Pathogenic Strains

To elucidate the optimal growth temperatures of the three strains, a series of experiments were conducted. Firstly, mycelial blocks from the edge of the colony were punched using a 7 mm sterile punch and transferred to the center of PDA culture medium plates. These plates were then incubated in dark incubators set at temperatures ranging from 5 °C to 40 °C, with intervals of 5 °C (5 °C, 10 °C, 15 °C, 20 °C, 25 °C, 30 °C, 35 °C, and 40 °C). There are 9 samples for each treatment, repeated three times, and colony diameter was measured after 7 days (or 5 days for strains A1 and D2) using the cross method. For spore germination rate analysis, a tray lined with two layers of water-soaked gauze and several 35 mm Petri dishes was prepared. Into each Petri dish, 1 mL of spore suspension and 1 mL of 1% glucose solution were added and mixed thoroughly. The lids were then placed onto the dishes to create a hydrophobic environment, and the tray was sealed with plastic wrap. Subsequently, the tray was placed in incubators set at different temperatures and incubated in darkness for 6 h. Following incubation, microscopic observations were conducted to determine the spore germination rate. A total of 50 spores were counted in each field of view, with each culture dish inspected nine times under the microscope. The spore germination rate (%) was calculated using the formula: (number of germinated spores/total number of spores) × 100.

To investigate the growth characteristics of the three types of strains under varying lighting conditions, mycelial blocks were prepared as previously described. These blocks were placed in Petri dishes, which were then incubated under three different lighting regimes: continuous light (24 h of light), 12 h of light followed by 12 h of darkness and continuous darkness (24 h of dark). The light intensity was consistently set to 1500 lx, and the temperature was maintained at a constant 25 °C. These conditions aimed to assess the impact of light on the growth of the strains over a specified period.

### 4.5. BIOLOG Phenotype Microarray Characteristic Analysis

The biological metabolic characteristics of the pathogenic strains were analyzed using the BIOLOG phenotype microarray technology. The strains were cultured in PDB medium at 25 °C with shaking at 180 rpm for 7 days. After incubation, the cultures were filtered through three layers of lens paper and centrifuged to remove the supernatant. The resulting precipitate was washed three times with sterile water. The washed precipitate was then added to the FF-IF inoculum solution and mixed thoroughly to prepare the final conidial suspension mother liquor. The turbidity meter was adjusted to 100% T (the standard concentration unit of the BIOLOG phenotype microarray technology) using the original FF-IF as a control. Small amounts of the conidial suspension mother liquor were added to the control inoculum solution incrementally, mixed gently, and the T value was measured until it was adjusted to 60–70% T. The configuration of the BIOLOG inoculum solution is detailed in Table S2. For carbon source utilization analysis, the bacterial suspension was poured into a V-shaped sampling tank and inoculated into the PM1 and PM2A plates using a pipette (100 μL per well). For nitrogen sources and biosynthetic pathway substances utilization, the bacterial suspension was mixed with 0.375 mL of solution A and 1 mL of solution B, shaken well, and then inoculated into the PM3B and PM5 plates. For phosphorus and sulfur sources utilization, the bacterial suspension was mixed with 0.375 mL of solution A, shaken well, and then inoculated into the PM4 plate. For analyzing metabolism under different osmotic pressures, ionic strengths, and pH conditions, the bacterial suspension was mixed with 0.375 mL of solution A and 1 mL of solution C, shaken well, and inoculated onto the PM9 plate.

Subsequently, the inoculated PM plates were sealed and placed in the Biolog system incubator. The temperature was set to 25 °C, and the incubation period was set to 7 days, with OmniLog 2.4 parameters configured to read every 15 min.

For data analysis, the Date File Converter software was used to convert the data file into OKA format. This file was then imported into Biolog OL_FM_2_1.20.02software and converted into DLB format. The Biolog OL_PR_2_1.20.02 software was subsequently used to import the DLB file of each strain for data analysis and parameter comparison. The analysis parameter was set to Area Peak Area, with the metabolic capacity of the strain being proportional to the peak area value. Material utilization maps from different PM plates were saved in JPEG format, and the corresponding values were exported in xlsx format. These data were then used to generate heat maps for comparison using RStudio.2021 software.

### 4.6. In Vitro Screening of Fungicides Against Three Strains

To screen for chemical agents that can effectively inhibit the growth of the three pathogenic strains, 16 chemical agents were selected (see Table S3 for detailed information). Approximately 0.1 g of each original drug was weighed into a test tube. An appropriate amount of DMSO was added to dissolve the original drugs (triadimefon was dissolved in absolute ethanol, and carbendazim in 0.1% hydrochloric acid aqueous solution). These solutions were diluted to a concentration of 1000 μg/mL with sterile water, forming the pharmaceutical mother solutions. The mother solutions were then added to PDA plates to prepare a final concentration of 10 μg/mL. Using a 7 mm hole punch, strains cultured on PDA medium for 14 days were sampled to obtain several fungal plugs. The plugs were placed, mycelium side down, at the center of the drug-containing plates. The plates were incubated in the dark at 25 °C for 1 week, after which the diameter of the mycelial growth was measured. As a control, fungal plugs were inoculated on plates with the same volume of the corresponding organic solvent under identical conditions. The antibacterial rate of each agent was calculated using the following formula:

Inhibition Rate (%) = (D_control − D_treatment)/D_control × 100.

Preliminary screening considered a percent inhibition greater than 50% as the standard for effectiveness. There are 9 samples for each treatment, repeated three times.

Following the aforementioned screening, effective agents were selected for different strains, and these agents underwent subsequent mycelial toxicity and spore germination toxicity tests. The mycelial toxicity test followed the procedure described previously. The optimal fungicides and their concentration settings for different strains are outlined in Table S4. For the spore germination toxicity test, a spore suspension of 1 × 10^5^ spores/mL was prepared. Different concentration gradients of pharmaceutical solutions (refer to Table S5 for concentrations of different agents used for different strains) were also prepared. Each test involved taking 1 mL of the spore suspension and 1 mL of the respective pharmaceutical solution, mixing them well, and adding the mixture to a 35 mm Petri dish. The Petri dishes were placed in a 25 °C incubator with dark moisture and incubated for 6 h using the hydrophobic environment method.

### 4.7. In Vivo Screening of Fungicides on Plum Fruits

To assess the in vivo effects of fungicides, we first evaluated their protective effect. Three fungicide preparations with better antifungal effects on strain DW from the previous tests were selected. Three agents’ solutions with different concentrations were prepared using sterile water (The concentrations of chemicals are detailed in Table S6). Fresh plum fruits were washed and disinfected, then affixed to a 35 mm Petri dish. Using sterilized insect needles, 6 micro-injury holes were made within a 5 mm range on both sides of the fruit. The treated pharmaceutical solutions were evenly applied by spraying onto the fruit surface. Organic solvents of the same concentration were used as a blank control. After spraying, the fruits were transferred to an ultra-clean workbench for air blowing to dry the surface. Once dry, they were placed in a tray for moisturizing culture, sealed with plastic wrap, and incubated in a 25 °C incubator for 24 h in the dark. On the second day, the plastic wrap was removed, and the bacterial cake of the strain cultured on PDA for 14 days was inoculated into the micro wounds (puncher D = 5 mm) on both sides of the fruit. The bacterial cake was also inoculated onto fruits sprayed with organic solvents, while fruits sprayed with organic solvents and inoculated with PDA culture medium without bacteria served as controls. The fruits were resealed with plastic wrap and cultured in a 25 °C incubator for 7 days, after which the control effect was measured. There are 9 samples for each treatment, repeated three times.

For the therapeutic effect, only the order of spraying chemicals and inoculating bacteria differed. After disinfecting and slightly damaging the plum fruit, bacteria cake inoculation was performed first, followed by incubation for 24 h. Then, the chemicals were sprayed, and the control effect was measured after continuing to culture for 7 days. The prevention and therapeutic effect formula were calculated as follows:

Inhibition Rate (%) = (S_control − S_treatment)/S_control × 100.

### 4.8. Statistical Analysis

All experiments described in this study were conducted with a minimum of three replicates to ensure statistical robustness. The data are expressed as mean values ± standard deviation. Statistical analyses were carried out using SPSS software (version 23.0). Student’s *t*-test was employed for comparing two sets of data (* 0.01 < *p* < 0.05, ** 0.001 < *p* < 0.01, *** *p* < 0.001), while one-way analysis of variance (ANOVA) was utilized for comparing multiple groups of data, followed by post hoc LSD test (*p* < 0.05) for pairwise comparisons.

## Figures and Tables

**Table 1:** Control effect of three chemical fungicides on strain DW on plum.

Fungicides	Concentration (μg/mL)	Lesion Area (mm^2^)	Therapeutic Effect (%)	Lesion Area (mm^2^)	Protective Effect (%)
50% Fluazinam SC	10	134.00 ± 42.76 a	40.00	103.33 ± 5.77 a	41.73
	25	91.67 ± 6.35 b	58.27	69.33 ± 4.62 b	60.90
	50	32.00 ± 6.08 c	85.43	26.67 ± 2.89 c	84.96
	80	19.00 ± 8.67 c	91.35	7.00 ± 1.73 d	96.06
45% Prochloraz–manganese EW	5	180.67 ± 1.15 a	17.75	148.00 ± 6.93 a	19.82
	10	132.67 ± 19.63 b	39.61	70.00 ± 11.85 b	60.34
	25	113.33 ± 5.77 b	48.41	36.33 ± 9.81 c	79.51
	50	12.00 ± 3.00 c	94.54	9.33 ± 1.15 d	94.74
30% Pyraclostrobin SC	0.5	140.00 ± 13.86 a	36.27	116.67 ± 5.77 a	34.21
	1	86.67 ± 5.77 b	60.55	66.67 ± 4.62 b	62.41
	5	37.67 ± 3.79 c	82.85	28.33 ± 2.89 c	84.02
	10	0.00 ± 0.00 d	100.00	0.00 ± 0.00 d	100.00

Note: the letters behind the values showed significant differences between treatments (*p* < 0.05).

## Data Availability

If you are interested in the strains or any data in this article, please feel free to contact the corresponding author (Guanhua Ma, nikemgh@swu.edu.cn).

## Supplementary Materials

The following supporting information can be downloaded at: https://www.mdpi.com/article/10.3390/plants15030369/s1, Figure S1: Comparison of pathogenicity of three strains on leaves and fruits. Table S1: Primers used in this article. Table S2: Biolog inoculum. Table S3: Preliminary screening of 13 chemical fungicides for 3 strains. Table S4: Fungicide concentration of strain mycelium toxicity test. Table S5: Fungicide concentration of strain conidium toxicity test. Table S6: The concentration of fungicides in vitro fruit fungicide test. Table S7: Preliminary screening of 13 chemical fungicides for 3 strains. Table S8: Inhibitory effects of 6 fungicides on the mycelial growth of strain DW. Table S9: Inhibitory effects of 6 fungicides on spore germination of strain DW. Table S10: Inhibitory effects of 7 fungicides on mycelial growth of strain A1. Table S11: Inhibitory effect of 8 fungicides on spore germination of strain A1. Table S12: Inhibitory effects of 5 fungicides on mycelial growth of strain D2. Table S13: Inhibitory effects of 5 fungicides on spore germination of strain D2.

## References

[1] Fanning K.J., Topp B., Russell D., Stanley R., Netzel M. (2014). Japanese plums (*Prunus salicina* Lindl.) and phytochemicals—Breeding, horticultural practice, postharvest storage, processing and bioactivity. J. Sci. Food Agric..

[2] Igwe E.O., Charlton K.E. (2016). A systematic review on the health effects of Plums (*Prunus domestica* and *Prunus salicina*). Phytother. Res..

[3] Li Y., Deng W., Wu L., Chen S., Zheng Z., Song H. (2023). Anti-inflammatory effects of polyphenols from plum (*Prunus salicina Lindl*) on RAW264.7 macrophages induced by monosodium urate and potential mechanisms. Foods.

[4] Lea M., Ibeh C., desBordes C., Vizzotto M., Cisneros-Zevallos L., Byrne D., Okie W., Moyer M.P. (2008). Inhibition of growth and induction of differentiation of colon cancer cells by peach and plum phenolic compounds. Anticancer. Res..

[5] Noratto G., Porter W., Byrne D., Cisneros-Zevallos L. (2009). Identifying peach and plum polyphenols with chemopreventive potential against estrogen-independent breast cancer cells. J. Agric. Food Chem..

[6] Vizzotto M., Porter W., Byrne D., Cisneros-Zevallos L. (2014). Polyphenols of selected peach and plum genotypes reduce cell viability and inhibit proliferation of breast cancer cells while not affecting normal cells. Food Chem..

[7] Noratto G., Martino H.S.D., Simbo S., Byrne D., Mertens-Talcott S.U. (2015). Consumption of polyphenol-rich peach and plum juice prevents risk factors for obesity-related metabolic disorders and cardiovascular disease in Zucker rats. J. Nutr. Biochem..

[8] Shukitt-Hale B., Kalt W., Carey A.N., Vinqvist-Tymchuk M., McDonald J., Joseph J.A. (2009). Plum juice, but not dried plum powder, is effective in mitigating cognitive deficits in aged rats. Nutrition.

[9] Pallas V., Aparicio F., Herranz M.C., Amari K., Sanchez-Pina M.A., Myrta A., Sanchez-Navarro J.A. (2012). Ilarviruses of *Prunus* spp.: A continued concern for fruit trees. Phytopathology.

[10] Lu M.M., Ma L.A., Tang L.H., Chen X.L., Guo T.X., Mo J.Y., Huang S.P., Li Q.L. (2022). First report of anthracnose of Sanhua Plum caused by *Colletotrichum aeschynomenes* in Guangxi, China. Plant Dis..

[11] Chen X., Zhang M., Tang L., Huang S., Guo T., Li Q. (2023). Screening and characterization of biocontrol bacteria isolated from Ageratum conyzoides against Collectotrichum fructicola causing Chinese plum (*Prunus salicina* Lindl.) anthracnose. Front. Microbiol..

[12] Najafiniya M., de Farias A.R.G., Armand A., Jungkhun N., Jayawardena R. (2024). Characterization of *Colletotrichum* species obtained from citrus in northern Thailand and introducing a new host record for *C. plurivorum*. Plant Pathol..

[13] Dean R., Van Kan J.A.L., Pretorius Z.A., Hammond-Kosack K.E., Di Pietro A., Spanu P.D., Rudd J.J., Dickman M., Kahmann R., Ellis J. (2012). The Top 10 fungal pathogens in molecular plant pathology. Mol. Plant Pathol..

[14] Chang T., Hassan O., Jeon J.Y., Kim C.H., Lee D.M., Kim J.S., Kang E.C., Kim J. (2023). *Colletotrichum* diversity within different species complexes associated with fruit anthracnose in South Korea and their fungicides in-vitro sensitivity. Res. Plant Dis..

[15] Liu F., Ma Z.Y., Hou L.W., Diao Y.Z., Wu W.P., Damm U., Song S., Cai L. (2022). Updating species diversity of *Colletotrichum*, with a phylogenomic overview. Stud. Mycol..

[16] Deng X.Q., Guo Y., Zhang T.X. (2005). Biological Characteristics of the Pathogenic Fungus Causing anthracnose of Prunus Americana. Chin. Agric. Sci. Bull..

[17] Zhou Z.S., Xu C.N., Wu Y.X., Chi M.F., Zhang H.J., Ding Y.J. (2011). Identification of the Pathogen Causing Anthracnose on *Cerasus humilis* (Bge.) Sok. Acta Phytopathol. Sin..

[18] Wang Q., Bi Y.Q., He C.X., Guo Z.X., Zhong D.W., Li J.H., Chen Q.Z., You J.H., Huang Z.H., Li J.B. (2021). Disease Survey and Pathogen Identification of Anthracnose on ‘Banbianhong’ Plum. Chin. Agric. Sci. Bull..

[19] Hassan O., Lee Y.S., Chang T. (2019). Colletotrichum Species Associated with Japanese Plum (*Prunus salicina*) Anthracnose in South Korea. Sci. Rep..

[20] Summerell B.A., Laurence M.H., Liew E.C.Y., Leslie J.F. (2010). Biogeography and phylogeography of Fusarium: A review. Fungal Divers..

[21] Lin Z., Que Y., Liu P., Huang Y., Zhang M. (2014). Research Progress of Plant Fusarium Phytopathogen. Sugar Crops China.

[22] Dinh T.L., Hyorim C., Yunhee C., Anbazhagan M., Daseul L., SeungBeom H. (2023). Re-identification of *Colletotrichum acutatum* Species Complex in Korea and Their Host Plants. Plant Pathol. J..

[23] Usman H.M. (2021). Fungicide Resistance in Peach Anthracnose Fungi Colletotrichum Species in China. Ph.D. Thesis.

[24] Xia J.W., Sandoval-Denis M., Crous P.W., Zhang X.G., Lombard L. (2019). Numbers to names—Restyling the *Fusarium incarnatum*-*equiseti* species complex. Persoonia.

[25] Ramdial H., Latchoo R.K., Hosein F.N., Rampersad S.N. (2017). Phylogeny and Haplotype Analysis of Fungi Within the *Fusarium incarnatum*-*equiseti* Species Complex. Phytopathology.

[26] Syafiqa P., Ani W., Arif W., Haruhisa S., Achmadi P. (2022). Development of PCR-RFLP Technique for Identify Several Members of *Fusarium incarnatum-equiseti* Species Complex and *Fusarium fujikuroi* Species Complex. Plant Pathol. J..

[27] Lu M., Ma L., Tang L., Chen X., Guo T., Huang S., Li Q. (2023). Identification of the species of Fusarium causing plum leaf blight. Acta Phytopathol. Sin..

[28] Araújo M.B.M., Moreira G.M., Nascimento L.V., de Almeida Nogueira G., Nascimento S.R.d.C., Pfenning L.H., Ambrósio M.M.d.Q. (2020). Fusarium rot of melon is caused by several Fusarium species. Plant Pathol..

[29] Zhu S. (2021). Extraction and purification of β-arbutin from pear leaves and study on the characteristics of phospholipid complex. Master’s Thesis.

[30] Hu Y. (2021). Extraction and Determination of Arbutin in Rhodiola. Shanghai Chem. Ind..

[31] Liu X., Wang X., Liu X.T., Wang X.X. (2022). Research progress on the pharmacological action and mechanism of arbutin. Food Ferment. Ind..

[32] Zhou N., Wu Y., Cai Y., Min S. (2022). Coupling Mechanism of Phosphorus and Nitrogen, Carbon Cycles in Critical Zone of Wetland. J. Earth Sci. Environ..

[33] Zhu W., Zhou M., Xiong Z., Peng F., Wei W. (2017). The cAMP-PKA Signaling Pathway Regulates Pathogenicity, Hyphal Growth, Appressorial Formation, Conidiation, and Stress Tolerance in *Colletotrichum higginsianum*. Front. Microbiol..

[34] Wang D., Qu Y., Gao P. (1996). Studies on the Regulation of Cellulase System by ATP and cAMP in Mycelial Fungi. Acta Microbiol. Sin..

[35] Babalola O.O. (2007). Pectinase and cellulase enhance the control of *Abutilon theophrasti* by *Colletotrichum coccodes*. Biocontrol Sci. Technol..

[36] Wang S., Gong J., Gao A., Zhao T., Luo R., Wang S., Ni T., Chen Q. (2010). Pharmaco-Toxicological Study of Preservative. J. Anhui Agric. Sci..

[37] SooYeon A., Seyoung J., Sudheer P.D.V.N., KwonYoung C. (2021). Microbial Production of Melanin Pigments from Caffeic Acid and L-Tyrosine Using *Streptomyces glaucescens* and FCS-ECH-Expressing *Escherichia coli*. Int. J. Mol. Sci..

[38] Eisenman H.C., Greer E.M., McGrail C.W. (2020). The Role of Melanins in Melanotic Fungi for Pathogenesis and Environmental Survival. Appl. Microbiol. Biotechnol..

[39] Zhen X., Lin X., Yang Z., Ye J., Lu J., Liang Y. (2024). Research progress on tea anthracnose pathogen infection. J. China Agric. Univ..

[40] Chantal F., Marta M., Lillian B., Inês D.M., Ferreira I.C.F.R., Piedade A.P., Casadevall A., Gonçalves T. (2021). Pyomelanin synthesis in *Alternaria alternata* inhibits DHN-Melanin synthesis and decreases cell wall chitin content and thickness. Front. Microbiol..

[41] Yang Y.F., Zhan H.X., Chen Y.T., Lu B.H., Liu L.P., Gao J. (2023). Research Progress on the Pathogenic Mechanisms of Anthracnose Pathogens in *Panax* Species. J. Jilin Agric. Univ..

[42] Du L.F., Zeng Q., Xu J., Zhang S.Y., Yu G.X., Guo T.R., Mo Y.W. (2022). Identification, Biological Characteristics and In Vitro Fungicide Screening of the Pathogen Causing *Fusarium* Fruit Rot on Hami Melon. J. Fruit Sci..

[43] Yu Q.T., Yao T.S. (2018). Research Progress on *Fusarium* Root Rot of Tobacco. J. Anhui Agric. Sci..

[44] Qian H.W., Xu P.C., Chi M.Y., Huang J.G. (2017). Combined Infection of *Fusarium oxysporum* and *Alternaria tenuissima* Causes Sweet Potato Stem Blight. Acta Phytophylacica Sin..

[45] Wang S., Zhang X., Zhang Z., Chen Y., Tian Q., Zeng D., Xu M., Wang Y., Dong S., Ma Z. (2023). Fusarium-produced vitamin B(6) promotes the evasion of soybean resistance by *Phytophthora sojae*. J. Integr. Plant Biol..

[46] Li Y.Y., Wu M., Wang X., Gu H.P., Chen X., Cui X.Y. (2024). Pathogen Identification of Soybean Anthracnose and Evaluation of Soybean Germplasm Resistance. Acta Phytopathol. Sin..

[47] Yang L., Bai Y., Yang L.J., Bai Y.L. (2012). Strobilurin fungicide—Pyraclostrobin. Mod. Agrochem..

[48] Hu S., Zhang S., Xiao W., Liu Y., Yu H., Zhang C. (2023). Diversity and characterization of resistance to pyraclostrobin in *Colletotrichum* spp. from Strawberry. Agronomy.

[49] Zhang K. (2023). Preparation of 20% fluazianm pyralostrobin suspension concentrate and its control effect on anthracnose of pepper. Master’s Thesis.

[50] Zhang Q. (2022). Analysis of main pesticide damage of crops and its countermeasures. J. Smart Agric..

