# Supplementary material for: Identification and Management of a Novel Brown Spot Disease in Plums (Prunus salicina Lindl.)
Source: Plants (Basel). 2026 Jan 24;15(3):369. doi: 10.3390/plants15030369 (PMC12899390; doi:10.3390/plants15030369)
Supplement: Supplementary file 1 [file plants-15-00369-s001.zip › plants-4072466-supplementary.pdf]

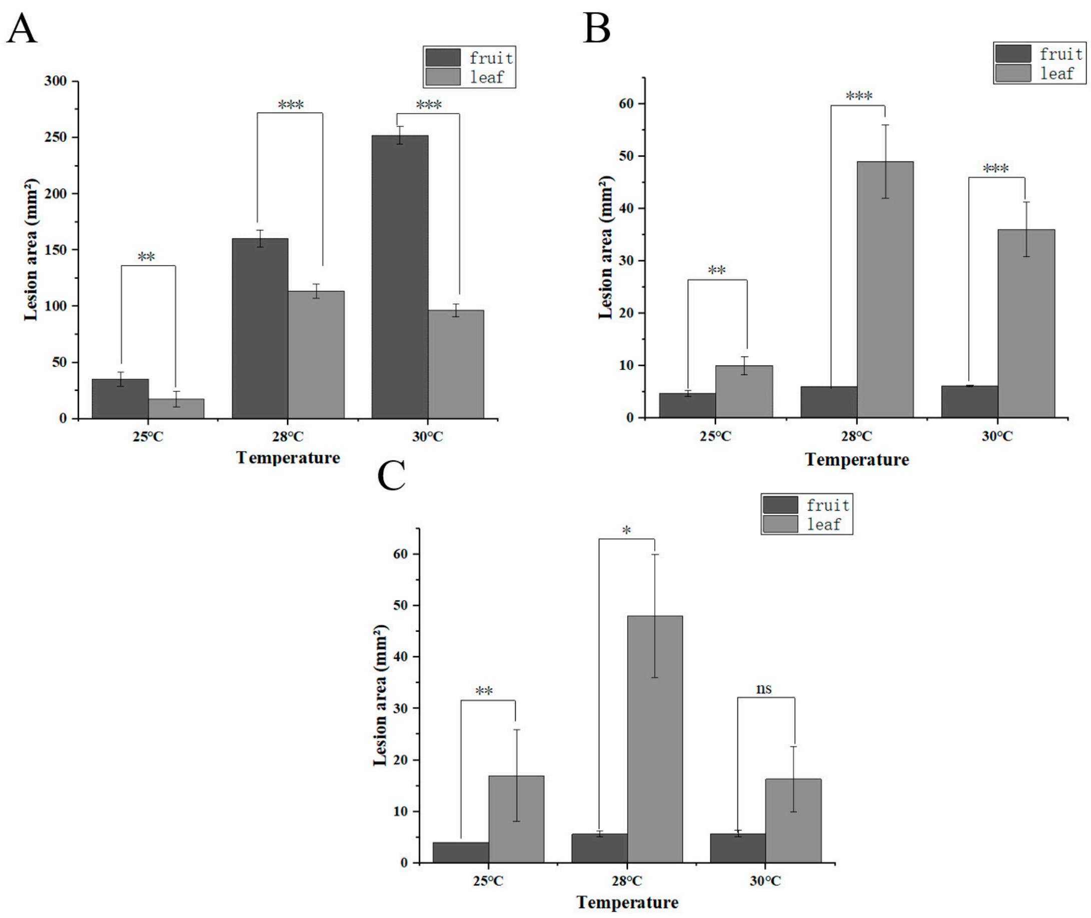

Figure S1 Comparison of pathogenicity of three strains on leaves and fruits. A. The pathogenicity of strain DW on fruits and leaves was different. B. The pathogenicity of strain A1 on fruits and leaves was different. C. The pathogenicity of strain D2 on fruits and leaves was different.

Table S1 Primers used in this article

Gene	Primers	Primers sequence 5'-3'	Annealing temperature
ITS	ITS1	TCCGTAGGTGAACCTGCGG	55℃
	ITS4	TCCTCCGCTTATTGATATGC	
ACT	ACT-512F	ATGTGCAAGGCCGGTTTCGC	55℃
	ACT-783R	TACGAGTCCTTCTGGCCCAT	
CHS-1	CHS-79F	TGGGGCAAGGATGCTTGGAAGAAG	55℃
	CHS-354R	TGGAAGAACCATCTGTGAGAGTTG	
GAPDH	GDF	GCCGTCAACGACCCCTTCATTGA	55℃
	GDR	GGGTGGAGTCGTACTTGAGCATGT	
TUB2	T1	AACATGCGTGAGATTGTAAGT	55℃
	βt2b	ACCCTCAGTGTAGTGACCCTTGGC	
RPB2	RPB2-5F2	GCCTTCTTCTG(G/A)TC(T/A)CCCC	64℃
	RPB2-7CR	CCCATR(A/G)GCTTGY(C/T)TTR(A/G)CCCAT	
EF-1α	EF-1	ATGGGTAAGGAGGACAAGAC	58℃
	EF-2	GGAAGTACCAGTGATCATGTT	
CAM	CAM-F	GTCAATCTAGGCGCGCCTA	55℃
	CAM-R	CGAACTCGTTGTCTGTAGA	

Table S2 Biolog inoculum

Name of reagent	Ingredient
A solution	57.664 glucose，100 mL distilled water
B solution	0.817 g Anhydrous potassium dihydrogen phosphate，0.341 g Sodium sulfate，100 mL distilled water
C solution	8.040 g Yeast Nitrogen Base，100 mL distilled water

Note: The above solutions need to be filtered with a 0.22 μm pore size microporous filter membrane and sterilized.

Table S3 Preliminary screening of 13 chemical fungicides for 3 strains

Mode of action	Chemical structure	Active principle	Percent	Manufacture
Inhibition of lipid and membrane synthesis	Dicarboxyimide	Iprodione	95%	Hubei Kangbaotai Fine Chemical Co., Ltd.
	Organophosphorou	Tolclofos-methyl	97%	Tianjin Hanbang Plant Protectant Co., Ltd.
Inhibition of sterol biosynthesis in membranes	Imidazoles	Prochloraz-manganese	96%	Tianjin Hanbang Plant Protectant Co., Ltd.
		Triflumizole	97%	Tianjin Hanbang Plant Protectant Co., Ltd.
	Triazoles	Difenoconazole	95%	Hubei Kangbaotai Fine Chemical Co., Ltd.
		Triadimefon	95%	Hubei Kangbaotai Fine Chemical Co., Ltd.
Inhibit respiratory function	Formamides	Thiofuramide	98%	Tianjin Hanbang Plant Protectant Co., Ltd.
	Methoxyacrylates	Pyraclostrobin	98%	Tianjin Hanbang Plant Protectant Co., Ltd.
	Dinitroanilines	Fluazinam	98%	Tianjin Hanbang Plant Protectant Co., Ltd.
Inhibition of signal transduction	Phenylpyrrole	Fludioxonil	98%	Tianjin Hanbang Plant Protectant Co., Ltd.
	Isoxazoles	Hymexazol	97%	Tianjin Hanbang Plant Protectant Co., Ltd.
Inhibition of mitosis and cell division	Thiocarbamates	Thiophanate-methyl	97.58%	Tianjin Hanbang Plant Protectant Co., Ltd.
Multi site joint activity	Dithiocarbamates	Mancozeb	80%	Hubei Kangbaotai Fine Chemical Co., Ltd.

Table S4 Fungicide concentration of strain mycelium toxicity test

Strain	Fungicides	Concentrations of effective components（μg/mL）				
DW	Fluazinam	2.560	0.640	0.160	0.040	0.010
	Pyraclostrobin	10.240	2.560	0.640	0.160	0.040
	Fludioxonil	5.120	1.280	0.320	0.080	0.020
	Prochloraz-manganese	0.200	0.100	0.050	0.025	0.0125
	Tolclofos-methyl	160.000	40.000	10.000	2.500	0.625
	Mancozeb	10.125	6.750	4.500	3.000	2.000
	Difenoconazole	5.120	1.280	0.320	0.080	0.020
A1	Fluazinam	10.240	2.560	0.640	0.160	0.040
	Pyraclostrobin	40.000	20.000	5.000	2.500	1.250
	Fludioxonil	10.240	5.120	2.560	1.280	0.640
	Prochloraz-manganese	5.120	2.560	1.280	0.640	0.320
	Tolclofos-methyl	40.000	20.000	10.000	5.000	1.250
	Triflumizole	5.120	2.560	1.280	0.320	0.080
	Triadimefon	40.000	20.000	10.000	5.000	2.500
	Hymexazol	40.000	20.000	10.000	5.000	1.250
D2	Tolclofos-methyl	80.000	40.000	20.000	10.000	5.000
	Fludioxonil	10.240	2.560	0.640	0.160	0.040
	Prochloraz-manganese	10.240	2.560	0.640	0.160	0.040
	Fluazinam	10.240	2.560	0.640	0.160	0.040
	Triflumizole	10.240	2.560	0.640	0.320	0.160

Table S5 Fungicide concentration of strain conidium toxicity test

Strain	Fungicides	Concentrations of effective components（μg/mL）				
DW	Fluazinam	0.500	0.400	0.200	0.150	0.0075
	Pyraclostrobin	0.100	0.050	0.010	0.005	0.001
	Fludioxonil	80.000	70.000	60.000	40.000	10.000
	Prochloraz-manganese	10.000	5.000	1.000	0.500	0.100
	Tolclofos-methyl	60.000	50.000	40.000	30.000	20.000
	Mancozeb	4.000	3.000	2.000	1.000	0.500
	Difenoconazole	10.000	5.000	1.000	0.500	0.050
A1	Fluazinam	1.000	0.500	0.100	0.050	0.010
	Pyraclostrobin	25.000	10.000	5.000	1.000	0.500
	Fludioxonil	5.000	1.000	0.500	0.100	0.050
	Prochloraz-manganese	10.000	5.000	1.000	0.500	0.100
	Tolclofos-methyl	60.000	50.000	40.000	30.000	20.000
	Triflumizole	10.000	5.000	1.000	0.500	0.100
	Triadimefon	60.000	50.000	40.000	30.000	20.000
D2	Hymexazol	50.000	40.000	30.000	20.000	10.000
	Tolclofos-methyl	60.000	40.000	30.000	20.000	10.000
	Fludioxonil	1.000	0.500	0.100	0.050	0.010
	Prochloraz-manganese	10.000	5.000	1.000	0.500	0.100
	Fluazinam	0.500	0.100	0.050	0.010	0.005
	Triflumizole	10.000	5.000	1.000	0.500	0.100

Table S6 The concentration of fungicides in vitro fruit fungicide test

Fungicides	Concentrations of effective components（μg/mL）			
50% Fluazinam SC	10.000	25.000	50.000	80.000
45% Prochloraz-manganese EW	5.000	10.000	25.000	50.000
30% Pyraclostrobin SC	0.500	1.000	5.000	10.000

Table S7 Preliminary screening of 13 chemical fungicides for 3 strains

Mode of action	Chemical structure	Active principle	Percent	Inhibition rate of colony growth (%)		
				DW strain	A1 strain	D2 strain
Inhibition of lipid and membrane synthesis	Dicarboxyimide	Iprodione	95%	21.20	32.34	18.29
	Organophosphorou	Tolclofos-methyl	97%	54.80	70.00	51.67
Inhibition of sterol biosynthesis in membranes	Imidazoles	Prochloraz-manganese	96%	100.00	100.00	79.88
		Triflumizole	97%	23.73	100.00	85.37
	Triazoles	Difenoconazole	95%	76.98	48.34	39.95
		Triadimefon	95%	30.51	68.00	26.52
Inhibit respiratory function	Formamides	Thiofuramide	98%	42.93	35.66	4.28
	Methoxyacrylates	Pyraclostrobin	98%	93.78	72.00	21.95
	Dinitroanilines	Fluazinam	98%	100.00	85.00	87.20
Inhibition of signal transduction	Phenylpyrrole	Fludioxonil	98%	89.62	78.34	67.68
	Isoxazoles	Hymexazol	97%	16.10	65.34	26.23
Inhibition of mitosis and cell division	Thiocarbamates	Thiophanate-methyl	97.58%	48.86	25.66	3.06
Multi site joint activity	Dithiocarbamates	Mancozeb	80%	70.34	45.00	21.35

Table S8 Inhibitory effects of 7 fungicides on the mycelial growth of strain DW

Fungicides	Regression equation	Correlation coefficient, *r*	EC_{50} (μg/mL)	95%CL (μg/mL)
Pyraclostrobin	y=0.7349x+5.2960	0.9727	0.3956	0.2306 ~ 0.6787
Fluazinam	y=1.7190x+5.7988	0.9901	0.3430	0.2571 ~ 0.4576
Fludioxonil	y=0.8501x+5.6398	0.9895	0.1768	0.1093 ~ 0.2860
Prochloraz-manganese	y=1.1542x+6.3433	0.9920	0.0686	0.0490 ~ 0.0960
Tolclofos-methyl	y=0.6485x+4.1807	0.9987	18.3358	10.0363 ~ 33.4985
Mancozeb	y=1.3458x+3.8052	0.9396	7.7226	5.8043 ~ 10.2748
Difenoconazole	y=0.5561x+5.1961	0.9974	0.4441	0.2223 ~ 0.8871

Table S9 Inhibitory effects of 7 fungicides on spore germination of strain DW

Fungicides	Regression equation	Correlation coefficient, *r*	EC_{50} (μg/mL)	95%CL (μg/mL)
Pyraclostrobin	y=1.0305x+7.8283	0.9727	0.0018	0.0012 ~ 0.0027
Fluazinam	y=0.4936x+5.7903	0.9600	0.0250	0.0106 ~ 0.0592
Fludioxonil	y=0.7580x+4.7858	0.9828	1.9170	1.1785 ~ 3.1184
Prochloraz-manganese	y=1.0538x+5.7182	0.9858	0.2082	0.1471 ~ 0.2946
Tolclofos-methyl	y=2.4613x+0.2054	0.9706	88.7107	62.8870 ~ 125.1384
Mancozeb	y=2.8160x+3.9974	0.9877	2.2701	1.6897 ~ 3.0498
Difenoconazole	y=0.6293x+5.7215	0.9319	0.0714	0.0397 ~ 0.1282

Table S10 Inhibitory effects of 8 fungicides on mycelial growth of strain A1

Fungicides	Regression equation	Correlation coefficient, *r*	EC_{50} (μg/mL)	95%CL (μg/mL)
Prochloraz-manganese	y=1.9178x+4.6665	0.9602	1.4924	1.1419 ~ 1.9384
Fluazinam	y=0.7688x+5.4054	0.9719	0.2970	0.1580 ~ 0.5580
Fludioxonil	y=1.0718x+4.4897	0.9842	2.9932	2.0631 ~ 4.3427
Triflumizole	y=1.3290x+5.1087	0.9964	0.8283	0.5694 ~ 1.2048
Triadimefon	y=0.8046x+4.2781	0.9816	7.8919	4.8315 ~ 12.8911
Tolclofos-methyl	y=0.8485x+4.0139	0.9959	14.5298	9.0556 ~ 23.3121
Hymexazol	y=0.9189x+4.6771	0.9855	2.2460	1.4194 ~ 3.5540
Pyraclostrobin	y=0.4935x+4.8874	0.9793	1.6609	0.7556 ~ 3.7836

Table S11 Inhibitory effect of 8 fungicides on spore germination of strain A1

Fungicides	Regression equation	Correlation coefficient, *r*	EC_{50} (μg/mL)	95%CL (μg/mL)
Fluazinam	y=1.1495x+5.8354	0.9714	0.1876	0.1179 ~ 0.2986
Fludioxonil	y=0.5605x+5.7147	0.9831	0.0531	0.0172 ~ 0.1638
Prochloraz-manganese	y=0.5991x+5.0444	0.9879	0.8431	0.3783 ~ 1.8791
Triflumizole	y=0.2629x+5.3926	0.9633	0.0321	0.0028 ~ 0.3711
Tolclofos-methyl	y=1.5349x+2.9255	0.9884	22.4672	16.2850 ~ 30.9965
Triadimefon	y=2.8059x+0.4144	0.9916	43.0791	36.0456 ~ 51.4850
Pyraclostrobin	y=0.6119x+5.6182	0.9903	0.0976	0.0353 ~ 0.2699
Hymexazol	y=2.2294x+2.6326	0.9925	11.5318	8.9803 ~ 14.8082

Table S12 Inhibitory effects of 5 fungicides on mycelial growth of strain D2

Fungicides	Regression equation	Correlation coefficient, *r*	EC_{50} (μg/mL)	95%CL (μg/mL)
Fluazinam	y=0.8056x+5.2835	0.9655	0.4447	0.2551 ~ 0.7751
Prochloraz-manganese	y=0.7848x+4.6823	0.9896	2.5398	1.4059 ~ 4.5880
Triflumizole	y=0.3612x+5.1073	0.9824	0.5045	0.1858 ~ 1.3701
Fludioxonil	y=0.8822x+5.2166	0.9652	0.5681	0.3574 ~ 0.9031
Tolclofos-methyl	y=0.7842x+4.3950	0.9803	5.9081	3.7337 ~ 93488

Table S13 Inhibitory effects of 5 fungicides on spore germination of strain D2

Fungicides	Regression equation	Correlation coefficient, *r*	EC_{50} (μg/mL)	95%CL (μg/mL)
Fluazinam	y=1.0246x+7.3650	0.9938	0.0049	0.0027 ~ 0.0091
Fludioxonil	y=1.1329x+6.6034	0.9659	0.0384	0.0232 ~ 0.0638
Prochloraz-manganese	y=0.7389x+5.5653	0.9915	0.1718	0.0799 ~ 0.3692
Triflumizole	y=0.4088x+5.2567	0.9808	0.2356	0.0672 ~ 0.8262
Tolclofos-methyl	y=1.1111x+3.3643	0.9989	29.6581	18.4947 ~ 47.5596
